# Fostering positive mental health outcomes in vulnerable children: Pathways to resilience after preterm birth

**DOI:** 10.1111/jcpp.70002

**Published:** 2025-07-16

**Authors:** E. Sabrina Twilhaar, Dieter Wolke

**Affiliations:** ^1^ Department of Psychology University of Warwick Coventry UK; ^2^ Division of Health Sciences, Warwick Medical School University of Warwick Coventry UK

**Keywords:** Resilience, mental health, preterm birth, protective factors, sex differences, adversity, longitudinal studies

## Abstract

**Background:**

Children born preterm (<37 weeks' gestation) are at increased risk of mental health problems, and their mental health outcomes have not improved in the past decades. This study aims to (1) determine the degree of mental health resilience in preterm‐born children; (2) identify modifiable factors at individual, parent–child, family, peer group, and neighbourhood levels associated with resilience; (3) explore differential effects of factors based on sex and contextual adversity.

**Methods:**

Preterm‐born children from the Bavarian Longitudinal Study (BLS; *n* = 574) born in Germany (1985–1986) and Millennium Cohort Study (MCS; *n* = 985) born in the UK (2000–2002) were assessed prospectively at 7 (MCS) or 8 (BLS) years. Resilience was defined as better‐than‐expected mental health outcomes, using a residuals approach. Potential promotive factors included (1) individual: self‐regulation, perceived competence, cognition; (2) parent–child relationships; (3) family: home environment, interparental relationship, social support, sibling relationships; (4) peers: bullying, friendships; and (5) neighbourhood characteristics. Associations between promotive factors and resilience were tested using regression‐based methods, with sex and contextual adversity (adverse life events, psychosocial stress, socioeconomic deprivation) as moderators and mediators.

**Results:**

The following factors were consistently (in both cohorts) associated with resilience: (1) individual: regulatory abilities, cognition; (2) parent–child: mother–child relationship; (3) family: authoritative and structured climate, interparental relationship; (4) peers: bullying. Regulatory abilities showed independent effect in both cohorts. Collectively, factors explained 30%–41% of the variance in resilience. Effects were similar across sex and contextual adversity, but promotive factors were less prevalent in boys and adverse contexts. Particularly in the UK, promotive resources were scarce amidst contextual adversity, which explained the lower resilience of children living in adversity.

**Conclusions:**

This study identified modifiable factors such as child self‐regulation, interparental relationships, and bullying that – if improved – have a high potential for improving mental health outcomes in preterm‐born children.

Most mental health problems have their onset before age 18 years (Solmi et al., 2022). A sizeable population that is easily identifiable at birth and at higher risk for mental health problems than the general population is preterm‐born children (<37 weeks' gestation) (Anderson et al., 2021). This at‐risk group comprises 10% of births globally each year, estimated at 13.4 million babies in 2020 (Ohuma et al., 2023). Incidence and survival rates of preterm birth have risen in high‐income countries over recent decades, suggesting a growing population of children vulnerable to mental health problems. Moreover, mental health outcomes of preterm‐born children show no signs of improvement (Larsen et al., 2023). Thus, preterm‐born children are an important target population for the prevention of mental health problems with significant potential impact.

Multiple pathways contribute to increased risks for mental health problems after preterm birth, including brain alterations, perinatal pain, early separation in the neonatal unit, postnatal stress, parental stress, and bullying victimisation (Montagna & Nosarti, 2016; Wolke, Baumann, Strauss, Johnson, & Marlow, 2015). Due to a predominantly biomedical focus in preterm birth research, the significant impact of social/environmental factors, particularly beyond the neonatal period, has long been overlooked. This is remarkable, considering the long‐standing recognition of the environment's crucial role in child development (Bronfenbrenner, 1977). More recent studies point to the importance of factors such as maternal sensitivity (Faure et al., 2017), maternal psychosocial distress (Lean et al., 2020), and the home environment (Treyvaud et al., 2012) for children's mental health outcomes after preterm birth. Moreover, most studies adopted a deficit model, focusing on risk factors for adverse outcomes in preterm‐born children. However, problems can be reduced or prevented not only by decreasing risk but also by strengthening factors associated with resilience, that is, protective and promotive factors. Resilience can be defined as “the capacity of a dynamic system to adapt successfully to challenges that threaten the function, survival, or development of the system” (Masten, 2021). Identifying modifiable factors at multiple systems, for example, at the individual, family or neighbourhood level, that promote resilience may provide levers to improve mental health complementing factors identified from deficit models. The narrow focus on risks, deficits and biomedical factors has neglected mechanisms contributing to adaptive outcomes. This leaves much potential for improving outcomes after preterm birth untapped. The need to broaden our view is evidenced by the lack of improvement in mental health outcomes of preterm‐born children in recent decades (Larsen et al., 2023) and limited evidence of long‐term effects of existing interventions (Herd, Whittingham, Sanders, Colditz, & Boyd, 2014; Khurana, Kane, Brown, Tarver, & Dusing, 2020).

Extensive research has provided a relatively consistent picture of multisystem factors contributing to resilience in non‐preterm child and adolescent populations (Fritz, De Graaff, Caisley, Van Harmelen, & Wilkinson, 2018; Masten, Lucke, Nelson, & Stallworthy, 2021). A few studies in preterm‐born children showed associations of parent–child relationships, parenting and family climate with mental health resilience (Neel et al., 2022; Taylor, Minich, Schluchter, Espy, & Klein, 2019), but no large‐scale studies including multiple modifiable multisystem factors are available. Resilience and factors that promote resilience may vary based on sex (Masten, 2015) and context (Ellis, Bianchi, Griskevicius, & Frankenhuis, 2017). Understanding such differential effects is particularly relevant for preterm‐born children given reports of increased mental health problems in boys (Twilhaar et al., 2022) and large social disparities in outcomes (Wolke, 2019).

The objectives of this study are first to determine the degree of mental health resilience in preterm‐born children. The degree of resilience will be based on the level of prematurity to determine which children have better‐than‐expected outcomes given their level of risk exposure (i.e. gestational age [GA] at birth) (Sapouna & Wolke, 2013). Second, we aim to identify modifiable multisystem factors associated with resilience. Empirically supported factors at the individual, parent–child, family, peer group and neighbourhood level preceding the outcome will be studied. Third, differential effects of protective/promotive factors according to sex and contextual adversity will be investigated. The study uses data from two population‐based cohorts: the Bavarian Longitudinal Study (BLS) and the Millennium Cohort Study (MCS).

## Methods

### Participants

BLS is a prospective geographical population‐based cohort of infants at neonatal risk born in Southern Bavaria, Germany in 1985–1986. A total of 7,505 infants were admitted to 16 children's hospitals within 10 days after birth, of whom 7,200 survived and 6,785 (94%) consented to participate and were assessed at birth, 5, 20, and 56 months of age. Of these infants, 2,803 were born preterm (<37 weeks' gestation). Due to funding limitations, a subsample was invited for continued follow‐up at 6 and 8 years, including 733 preterm‐born children, of whom 578 (79%) participated at 8 years with 574 (78%) children having mental health data available (Figure S1A).

MCS is a prospective population‐based cohort of children born between September 2000 and January 2002, living in the UK at age 9 months, and eligible for child benefits (i.e. universal benefit irrespective of income). Children from disadvantaged backgrounds and areas with more ethnic minorities were oversampled. Data up until 7 years were used in the present study. At 7 years, 17,031 families were contacted and 13,857 participated (81%). At 9 months, 1,478 preterm‐born (<37 weeks' gestation) children participated, of whom 1,028 (70%) participated at 7 years with mental health data available for 986 (67%) children (Figure S1B). BLS and MCS preterm samples are described in Table 1.

**Table 1:** Characteristics of the children included versus children who were not included in the study samples

	Bavarian Longitudinal Study				Millennium Cohort Study			
	Participants		Non‐participants^a^		Participants		Non‐participants^b^	
	CBCL data at 8 years		No CBCL data at 8 years		SDQ data at 7 years		No SDQ data at 7 years	
	*n* = 574		*n* = 159		*n* = 986		*n* = 492	
	*M* (*SD*) or *n* (%)	*M* (*SD*) or %	*M* (*SD*) or *n* (%)	*p*	*M* (*SD*) or *n* (%)	*M* (*SD*) or %	*M* (*SD*) or *n* (%)	*p*
	Unweighted	Weighted		Unweighted	Unweighted	Weighted		Unweighted
Demographic and perinatal characteristics								
Sex, male	303 (52.8%)	52.7%	81 (50.9%)	.680	523 (53.0%)	54.4%	271 (55.1%)	.459
Ethnicity								.**001**
White					837 (85.0%)	86.6%	373 (75.8%)	
Mixed					32 (3.2%)	4.5%	24 (4.9%)	
Indian					31 (3.1%)	2.0%	20 (4.1%)	
Pakistani or Bangladeshi					49 (5.0%)	3.4%	41 (8.3%)	
Black or Black British					31 (3.1%)	3.0%	29 (5.9%)	
Other					5 (0.5%)	0.6%	5 (1.0%)	
Gestational age, weeks	32.1 (2.8; 23–36)	32.0 (2.8)	30.5 (2.3; 25–36)	**<.001**	34.4 (2.4; 24.0–36.9)	34.4 (2.4)	34.1 (2.8; 24.3–36.9)	.**006**
Birth weight, g	1679.4 (604.4; 540–4,520)	1669.3 (603.8)	1437.9 (415.4; 630–2,800)	**<.001**	2369.2 (676.2; 390–4,420)	2359.5 (672.1)	2304.9 (722.4; 570–4,960)	.093
Multiple birth	101 (17.6%)	17.5%	28 (17.6%)	.997	156 (15.8%)	15.8%	82 (16.7%)	.677
Small for gestational age^c^	204 (35.5%)	35.7%	47 (29.6%)	.160				
Bronchopulmonary dysplasia^d^	183 (31.9%)	32.4%	87 (61.7%)	.076				
Postnatal sepsis	242 (42.2%)	42.7%	87 (54.7%)	.**005**				
Intraventricular haemorrhage				**<.001**				
Grade I, II	53 (9.2%)	9.5%	28 (17.9%)					
Grade III, IV	32 (5.6%)	5.8%	18 (11.5%)					
Parental characteristics								
Maternal age at birth, years	29.0 (5.2; 17–44)	29.0 (5.2)	28.9 (5.7; 16–42)	.736				
Maternal age at 9 months, years					29.8 (6.1; 15–52)	29.7 (6.2)	28.1 (6.3; 16–43)	**<.001**
Maternal tobacco use during pregnancy	99 (17.2%)	17.6%	42 (28.4%)	.**005**	256 (26.0%)	26.5%	135 (27.4%)	.544
Partner's tobacco use during pregnancy	196 (34.1%)	34.3%	67 (47.2%)	.**017**	267 (27.1%)	27.6%	114 (23.2%)	.105
Maternal alcohol use during pregnancy					94 (19.1%)	28.6%	262 (26.6%)	.**002**
Nationality, German	564 (98.3%)	97.9%	116 (73.0%)	**<.001**				
Family SES at birth^e^				.**002**				
Low	201 (35.0%)	35.5%	76 (50.0%)					
Intermediate	214 (37.3%)	37.3%	50 (32.9%)					
High	158 (27.5%)	27.1%	26 (17.1%)					
Maternal education at birth								
Tertiary education^f^	88 (15.3%)	15.1%	15 (9.9%)	0.076				
Paternal education at birth								
Tertiary education^f^	175 (30.5%)	30.0%	33 (22.1%)	.**020**				
Education level, highest between both parents								
Tertiary education^f^					351 (35.6%)	33.0%	101 (20.6%)	**<.001**
Marital status at birth, married or cohabiting	524 (91.3%)	91.2%	136 (90.1%)	.244				
Single parent at 9 months					36 (3.7%)	3.2%	23 (4.7%)	.343
Disabilities and early development								
Cerebral palsy at 4 years, any degree	58 (10.1%)	10.5%	20 (21.3%)	.**002**				
Neurosensory impairment at 4 years^g^	2 (0.3%)	0.4%	5 (5.9%)	**<.001**				
Hearing problems at 9 months					78 (7.9%)	8.7%	39 (7.9%)	0.991
Development score at 20 months^h^	97.6 (18.1; 14–120)	97.2 (18.4)	85.11 (27.80; 10–121)	**<.001**				

Differences are tested with Pearson's chi^2^, independent samples *t*‐test, or Fisher's exact test as appropriate. Bold *p*‐values indicate statistical significance at the *p* < .05 level.

**Figure 1:**
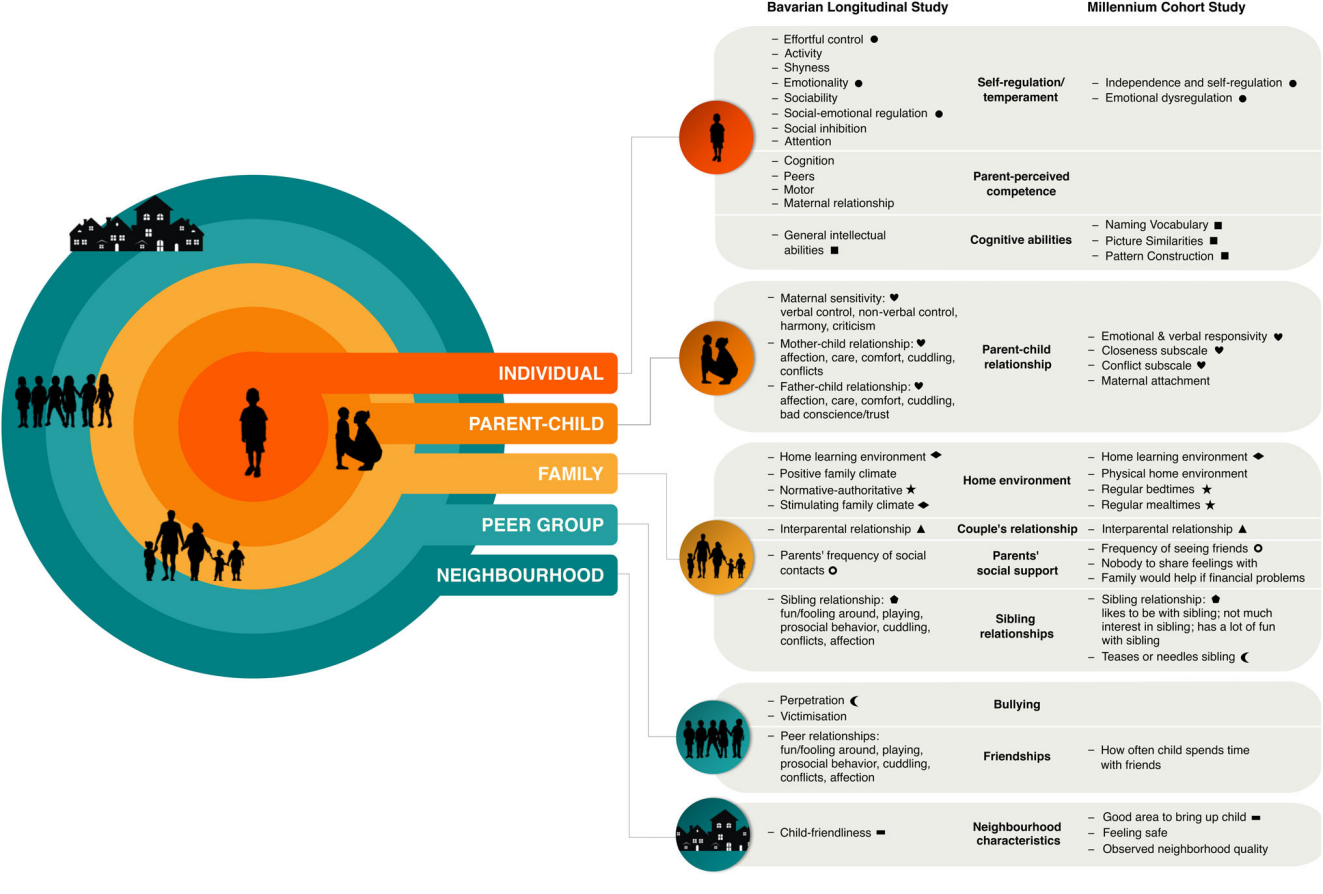
Protective/promotive and risk factors at each multisystem level as measured in the Bavarian Longitudinal Study and Millennium Cohort Study. Symbols indicate similar or overlapping constructs between cohorts. Detailed descriptions of constructs and how they were measured are provided in Table S1

^a^ Alive and selected for participation at 6 years but no CBCL data at 8 years due to ineligibility, dropout, or missing data (see Figure 1A).

^b^ Children with GA <37 weeks who participated at 9 months of age but who had no SDQ data at 7 years due to ineligibility, dropout, or missing data (see Figure 1B).

^c^ Birth weight <10th percentile for gestational age and sex.

^d^ Diagnosed from chest radiograph or need for supplemental oxygen for ≥28 days.

^e^ Categorised based on weighted composite score of maternal highest educational qualification, paternal highest educational qualification and occupation of the breadwinner.

^f^ Based on ISCED classifications (ISCED 5–7).

^g^ Hearing impairment (deaf, not corrected or deaf, insufficiently corrected) and/or blindness.

^h^ Total score on the Griffiths Scales of Mental Development, which measures the rate of development across locomotion, personal/social skills, hearing and speech, hand‐eye coordination, and performance.

### Procedures

The BLS was ethically approved by the Ethics Committee of the University of Munich Children's Hospital and the Bavarian Health Council (Landesärztekammer). All parents provided informed consent. For the MCS, ethical approval was obtained from the Multi‐centre Research Ethics Committees of London (3 and 5 years) and Yorkshire (7 years). Written consent was required from parents or guardians.

### Mental health resilience

Mental health was measured using the raw total scores of the Child Behaviour Checklist (CBCL) in the BLS, and of the Strengths and Difficulties Questionnaire (SDQ) in the MCS. Resilience was determined at 7 (MCS) and 8 (BLS) years of age using a residuals approach (Sapouna & Wolke, 2013). The residuals indicate to what extent children's observed scores deviate from model‐predicted scores based on GA (see Statistical analyses). Larger *negative* residuals reflect fewer problems than expected for GA, thus higher degrees of resilience.

### Protective/promotive factors

Factors were measured at different multisystem levels, depicted in Figure 1. Table S1 describes the measurement of each construct in detail.

### Contextual adversity

In BLS, a cumulative risk score of adversities across the neonatal period, 5, 20 and 56 months was calculated based on psychosocial stress (Psychosocial Stress Index), family adversity (Family Adversity Index), and socioeconomic deprivation (monthly family income < 25th percentile). In MCS, a cumulative risk score was based on adversity measured across 9 months, 3, and 5 years, including psychosocial stress (9 months: Rutter Malaise Inventory, 3 and 5 years: Kessler 6), adverse life events (single questionnaire items), and socioeconomic deprivation (area level: lowest decile of Index of Multiple Deprivation; family level: <60% of median poverty indicator OECD equivalence scale). Appendix S1 provides a detailed description.

### Statistical analyses

Appendix S1 provides a more detailed description of the statistical analyses. Using generalised additive models (GAM), the relation of gestational age and birthweight with mental health was captured by fitting non‐linear smooth functions with the *gam()* function of the *mgcv* package. This model was compared with a linear model using a corrected Akaike information criterion (AIC) (Wood, Pya, & Säfken, 2016).

Residuals of selected models were extracted as a measure of resilience and regressed on protective/promotive factors using Mplus and *lavaan*. Simple (unidimensional constructs) or multiple (multidimensional constructs) linear regression models were used for observed variables and structural equation modeling for latent constructs. Constructs were specified using confirmatory factor analysis. Indicators with factor loadings <0.40 were discarded. Appropriate fit was indicated by CFI ≥0.95 and RMSEA <0.06–0.08. Coefficient *H* was used to estimate reliability (Kalkbrenner, 2021). Missing data in protective/promotive factors (Table S2) were dealt with using full‐information maximum likelihood estimation.

To assess whether associations between resilience and protective/promotive factors varied across sex and contextual adversity, interaction terms were added to the models. For latent constructs, measurement invariance across sex was tested by constraining factor loadings to establish metric invariance. Mediation models were used to test (1) direct effects of sex and contextual adversity on resilience, (2) associations of sex and contextual adversity with protective/promotive factors and (3) indirect effects of sex and contextual adversity on resilience through the protective/promotive factor. Indirect effects are reported with percentile bootstrap confidence intervals to establish their statistical significance.

Finally, independent effects of protective/promotive factors were explored in a full regression model, including all observed and latent factors, adjusted for sex and contextual adversity. Factor scores were used for latent constructs after evaluating their potential bias (Appendix S1).

For MCS, weights accounted for clustering, stratification and non‐response (Centre for Longitudinal Studies, 2020). For BLS, inverse probability of censoring weights were calculated using variables that significantly differed between participants and non‐participants (Table 1) to account for selective dropout. All analyses were weighted and adjusted for the dependency of observations of multiples within the same family. To control the false discovery rate in multiple testing across models, a two‐stage linear step‐up procedure with *q* = 0.05 was used (Benjamini, Krieger, & Yekutieli, 2006).

## Results

### Data availability

Mental health data were available for 574 of 578 participants (99%) in BLS and for 985 of 1,028 participants (96%) in MCS. Even after weighting, children in the study samples had more favorable perinatal and social characteristics, were more often white, and had fewer disabilities (Table 1).

### Degree of resilience

#### Bavarian Longitudinal Study

**Figure 2:**
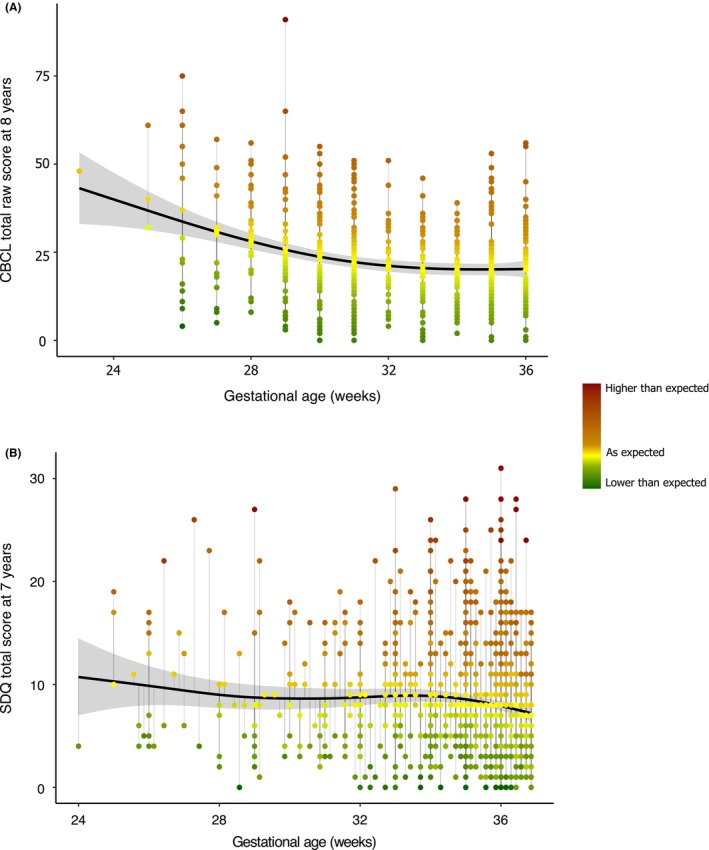
Non‐linear relations between gestational age and CBCL total raw scores at 8 years of age in the Bavarian Longitudinal Study (A) and between gestational age and SDQ total scores at 7 years of age in the Millennium Cohort Study (B). The dots represent the observed scores and are colored according to the direction and distance from the predicted score (i.e. the regression line) based on gestational age. Darker red means higher than expected scores (i.e. more problems). Darker green means lower than expected scores (i.e. less problems), indicating resilience

The relation between GA and CBCL scores was non‐linear (EDF = 2.68, *p* < .001). Based on AIC, adding birthweight did not improve model fit. Inclusion of additional neonatal factors did also not improve model fit (Appendix S2). As shown in Figure 2A, the decrease in CBCL scores with increasing GA was stronger in the lower GA range and plateaued towards 36 weeks. The green dots below the regression line represent children with, to a greater or lesser extent, lower CBCL scores (i.e. less problems) than expected given their GA. The further below the line, the higher the degree of resilience.

#### Millennium Cohort Study

GAM with a non‐linear smooth term for GA (EDF = 3.02, *p* = .14) and a linear term for birth weight (*B* = −0.00, *SE* = 0.00, *p* = .28) showed the best fit to the data (Figure 2B).

### Protective/promotive factors

#### Bavarian Longitudinal Study

Bivariate associations between protective/promotive factors and resilience at 8 years are shown in Table 2. At the individual level, effortful control (positive) and emotionality (negative) were most strongly associated with resilience. Additionally, attention abilities, parent‐perceived motor competence, and cognitive abilities were positively associated with resilience. At the parent–child level, the mother–child relationship quality was positively associated with resilience. Family factors that positively impacted resilience were interparental relationship quality and normative‐authoritative family climate. The effect of interparental relationship quality was stronger for boys than girls (Figure S2A, Table S3). Both bullying victimisation and perpetration were associated with lower resilience.

**Table 2:** Relation between each construct and mental health resilience tested in simple and multiple regression models in the Bavarian Longitudinal Study and the Millennium Cohort Study

System level	Instrument – Age of assessment	Construct, subscale, or item	Simple regression models				Multiple regression model^a^			
			*β*	*SE*	FDR‐corrected *p*	*R* ^2^	*β*	*SE*	FDR‐corrected *p*	Model *R* ^2^
Bavarian Longitudinal Study										
Individual	Emotionality, Activity, Sociability (EAS) – 6 years	Self‐regulation/temperament								
		Effortful control	−0.228	0.045	.**000**	.17	−0.160	0.047	.**007**	.30
		Activity	0.061	0.047	.349		0.066	0.045	.297	
		Shyness	−0.041	0.054	.567		0.029	0.051	.649	
		Emotionality	0.257	0.048	.**000**		0.201	0.045	.**000**	
		Sociability	−0.120	0.061	.147		−0.087	0.054	.241	
	Tester's Rating of Child Behaviour (TRCB) – 6 years	Social–emotional regulation	−0.030	0.047	.590	.00	0.029	0.041	.592	
	Team Rating of Child Attention (TEAM) – 6 years	Attention	−0.181	0.050	.**002**	.03	0.018	0.059	.750	
	Harter scales (parent‐rated) – 6 years	Parent‐perceived competence								
		Cognition	−0.119	0.055	.104	.08	−0.054	0.061	.517	
		Peers	−0.050	0.051	.486		−0.005	0.046	.815	
		Motor	−0.140	0.052	.**033**		−0.082	0.050	.237	
		Maternal relationship	−0.058	0.048	.382		−0.019	0.047	.718	
	Kaufman Assessment Battery for Children (KABC) – 6 years	Cognitive abilities	−0.154	0.053	.**021**	.02	0.045	0.064	.592	
	Social Inhibition Assessment – 6 years	Social inhibition	−0.046	0.048	.495	.00	−0.002	0.044	.846	
Parent–child	Assessment of Mother–Child‐Interactions with the Etch‐a‐Sketch (AMCIES) – 6 years	Maternal sensitivity	−0.115	0.049	.071	.01	−0.059	0.041	.305	
	Friendship and Family Interview, Card‐sorting task – 6 years	Mother–child relationship	−0.141	0.051	.**026**	.02	−0.163	0.038	.**000**	
		Father‐child relationship	−0.073	0.053	.322	.01	−0.131	0.036	.**002**	
		Sibling relationship	0.078	0.050	.259	.01	0.029	0.039	.575	
Family	Home Observation for Measurement of the Environment (HOME) – 6 years	Home learning environment	−0.059	0.058	.471	.00	−0.030	0.042	.575	
	Family Environment Scale (FES) – 6 years	Positive family climate	0.046	0.049	.495	.03	0.000	0.040	.851	
		Normative‐authoritative climate	−0.130	0.050	.**041**		−0.090	0.041	.100	
		Stimulating family climate	−0.091	0.046	.143		0.097	0.051	.158	
	Dyadic Adjustment Scale (DAS) – 6 years	Interparental relationship^b^	−0.251	0.057	.**000**	.06	−0.147	0.054	.**029**	
		Interparental relationship × sex^c^	−0.225	0.077	.**021**					
	Parent interview; social support – 6 years	Parents' frequency of social contacts	−0.055	0.047	.401	.00	−0.026	0.043	.631	
Peer group	Parent Interview – 6 years	Bullying								
		Perpetration	0.149	0.045	.**007**	.02	0.009	0.054	.785	
		Victimisation	0.185	0.044	.**000**	.03	0.115	0.050	.084	
	Friendship and Family Interview, Card‐sorting task – 6 years	Peer relationships	−0.097	0.050	.147	.01	−0.075	0.045	.226	
Neighbourhood	Parent interview; Living Situation Questionnaire – 6 years	Child‐friendliness neighbourhood	−0.105	0.051	.133	.01	−0.047	0.044	.451	
Millennium Cohort Study										
Individual	Child Social Behaviour Questionnaire (CSBQ) – 5 years	Independence and self‐regulation	−0.158	0.040	.**001**	.31	−0.127	0.038	.**007**	.38
		Emotional dysregulation	0.494	0.032	**<.001**		0.388	0.038	**<.001**	
	British Ability Scales (BAS) – 5 years	Naming Vocabulary	−0.106	0.042	.**049**	.06	0.004	0.035	.750	
		Picture Similarities	−0.020	0.049	.718		0.014	0.037	.715	
		Pattern Construction	−0.078	0.062	.369		−0.018	0.045	.734	
Parent–child	Home Observation for Measurement of the Environment (HOME)	Emotional and verbal responsivity^b^	−0.269	0.066	**<.001**	.07	−0.073	0.060	.405	
		Emotional & verbal responsivity × sex^c^	−0.203	0.064	.**012**					
		Emotional & verbal responsivity × context^c^	−0.191	0.061	.**012**					
	Child–Parent Relationship Scale (CPRS) – 3 years	Closeness subscale	−0.122	0.056	.102	.12	−0.014	0.041	.734	
		Conflict subscale	0.293	0.053	**<.001**		0.043	0.052	.575	
	Condon questionnaire – 9 months	Maternal attachment	−0.194	0.047	**<.001**	.04	−0.054	0.034	.246	
Family	HOME	Home learning environment	−0.132	0.053	.051	.02	−0.007	0.039	.781	
	HOME	Physical home environment^b^	−0.262	0.064	**<.001**	.07	−0.049	0.053	.495	
		Physical home environment × context^c^	−0.141	0.054	.**041**					
	Golombok Rust Inventory of Marital State – 5 years	Interparental relationship quality	−0.164	0.050	.**007**	.03	−0.009	0.045	.777	
	Parents' social support, parent interview	Nobody to share feelings with	0.119	0.055	.106	.05	0.027	0.048	.649	
		Family would help if financial problems	−0.087	0.066	.347		−0.013	0.050	.758	
		Frequency of seeing friends	−0.034	0.043	.555		0.000	0.035	.850	
	Family routines, parent interview	Regular bedtimes	−0.163	0.059	.**026**	.04	−0.076	0.049	.240	
		Regular mealtimes	−0.052	0.061	.529		0.041	0.043	.495	
	Sibling relationship, parent interview	Sibling relationship quality	−0.202	0.051	**<.001**	.08	−0.042	0.043	.471	
		Teases or needles sibling	0.191	0.044	**<.001**		−0.017	0.038	.710	
Peer group	Friendships, parent interview	How often child spend time with friends	0.087	0.044	.147	.00	0.061	0.038	.249	
Neighbourhood	Tester's observation	Observed neighbourhood quality	−0.252	0.048	**<.001**	.06	−0.080	0.044	.212	
	Parent interview	Good area to bring up a child	−0.147	0.048	.**012**	.04	−0.009	0.049	.758	
		Feeling safe^b^	0.019	0.047	.718		0.047	0.039	.382	
		Feeling safe × context^c^	0.529	0.199	.**036**					

Negative coefficients indicate that the higher the value of the promotive factor, the lower the residual value (i.e. higher degree of resilience). AMCIES, Assessment of Mother–Child‐Interactions with the Etch‐a‐Sketch; EAS, Emotionality, Activity, Sociability; FDR, false discovery rate; HOME, Home Observation for Measurement of the Environment; KABC, Kaufman Assessment Battery for Children. Bold *p*‐values indicate statistical significance at the *p* < .05 level.

^a^ Multiple regression models were adjusted for sex and contextual adversity.

^b^ The effect differed according to sex or contextual adversity, hence the main effect should not be interpreted.

^c^ Unstandardised interaction effects are shown (Preacher, 2003).

Exploration of independent effects of these factors on mental health resilience in a full regression model, adjusted for sex and contextual adversity, again revealed effects across multisystem levels (Table 2). Effortful control, emotionality, mother–child relationship, father‐child relationship and interparental relationship quality explained unique variance in resilience unshared by other factors.

#### Millennium Cohort Study

Factors across multisystem levels were associated with resilience (Table 2). At the individual level, these included independence and self‐regulation, emotional dysregulation, and naming vocabulary abilities. Conflict between mother and child, maternal attachment, and emotional and verbal responsivity were positively associated with resilience, although the latter only in boys (Figure S2B) and in high‐adversity contexts (Figure S2C). At the family level, interparental relationship quality, regular bedtimes, and sibling relationships were positively associated with resilience. The physical home environment was positively associated with mental health resilience in high‐ but not low‐adversity contexts (Figure S2D, Table 2). Finally, neighbourhood child‐friendliness (parent‐reported) and quality (assessor's observation) were positively associated with resilience. Increased feelings of safety in the neighbourhood as reported by parents were associated with higher mental health resilience when contextual adversity was low, but with lower mental health resilience when adversity was high (Figure S2E, Table 2). Post‐hoc adjustment for prenatal substance exposure did not alter the results in either cohort (Table S5).

In a full regression model (Table 2), independence and self‐regulation, and emotional dysregulation explained unique variance in mental health resilience unshared by other factors.

### Sex and contextual adversity

#### Bavarian Longitudinal Study

Mental health resilience and protective/promotive factors differed by sex (Figure S3A) and contextual adversity (Figure S3B). Resilience was lower in boys and children exposed to higher contextual adversity. A sex gap was found for some protective/promotive factors, generally showing lower levels in boys. Only attention skills mediated the relation between sex and resilience, indicating that boys' lower mental health resilience was partly explained by their poorer attention skills (Table S6). Higher contextual adversity was associated with lower levels of protective/promotive factors. Contextual adversity was associated with lower resilience through its negative effect on interparental relationship quality. Moreover, contextual adversity was associated with higher emotionality, which in turn was associated with lower resilience (Table S6).

#### Millennium Cohort Study

Like BLS findings, resilience was lower in boys (Figure S3C) and at higher contextual adversity levels (Figure S3D). Additionally, boys had lower independence and self‐regulation than girls and more often teased their siblings (Figure S3C), which both contributed to their lower resilience (Table S7). The levels of most protective/promotive factors were related to the level of contextual adversity (Figure S3D). Factors with a positive impact on resilience were lower when adversity levels were higher, whereas emotional dysregulation, mother–child conflict, and teasing siblings – associated with lower resilience – were higher in high‐adversity contexts. Indeed, emotional dysregulation, mother–child conflict, physical home environment, sibling relationship quality, teasing siblings and the observed neighbourhood quality mediated the negative relation between contextual adversity and resilience (Table S7).

### Integration of results

**Figure 3:**
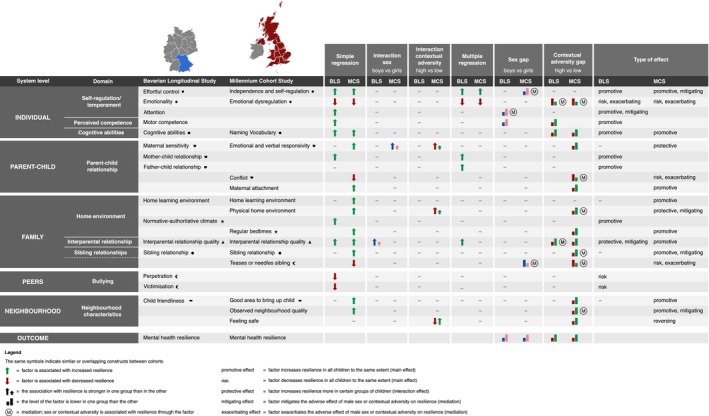
Integrative overview of statistically significant associations between multisystem factors and mental health resilience of preterm‐born children as tested in different models in the Bavarian Longitudinal Study and the Millennium Cohort Study

Figure 3 provides an integrative overview of the findings.

## Discussion

This study presents the first comprehensive analysis of a range of multisystem factors potentially promoting mental health resilience in preterm‐born children. As summarised in Figure 3, we found consistent evidence (i.e. in both cohorts) for six categories of factors contributing to resilience at early school age: (1) Regulatory abilities (*individual*), (2) cognitive abilities (*individual*), (3) mother–child relationship (*parent–child*), (4) home environment (*family*), (5) interparental relationship (*family*) and (6) bullying (*peer*, *sibling*). Additionally, evidence from either cohort supported the role of attention skills, parent‐perceived motor competence, maternal attachment, parental responsivity, physical home environment, home learning environment, sibling relationship quality and neighbourhood quality. Factors across all system levels pictured in Figure 1 contributed to resilience. Only for regulatory abilities was there consistent evidence for an independent effect over and above other protective, promotive and risk factors. While single factors exhibited small to medium effects, collectively, they explained 30%–41% of the variance in resilience. Effects of most factors were similar across sex and contextual adversity. However, promotive factors were less prevalent in boys and children facing higher contextual adversity, particularly in the UK cohort (MCS). In this cohort, the scarcity of promotive resources amidst contextual adversity explained the lower resilience of children facing higher levels of adversity.

Our results highlight the central role of regulatory abilities for mental health resilience, as emphasised by others (Daniel, Abdel‐Baki, & Hall, 2020; Fritz et al., 2018; Masten et al., 2021). Both cognitive (effortful control, attention) and emotional (emotionality, emotional dysregulation) aspects of self‐regulation were key for mental health resilience in preterm‐born children. Although regulatory abilities are usually measured at the individual level – inner layer of Figure 1 – they are reliant on robust contextual systems – outer layers of Figure 1 – to support adaptive behaviour (Ungar & Theron, 2020). Moreover, regulatory capacities are not merely determined by individual factors but are facilitated by the environment (McClelland et al., 2018). Parents can facilitate their development through three parenting dimensions: involvement, autonomy support and structure (Grolnick, Caruso, & Levitt, 2019). In our study, multiple factors related to these dimensions were associated with resilience, including relationship quality, responsivity, conflict and attachment at the parent–child level, and normative‐authoritative climate and regular bedtimes at the family level. These factors shared their influence on resilience with regulatory abilities and other factors, suggesting they are associated with resilience partly through their impact on regulatory abilities. In line with a diathesis‐stress model, low levels of responsive parenting adversely affected children's mental health in highly adverse contexts in MCS. Additionally, responsivity affected mental health resilience in preterm boys but not girls. Low responsivity was associated with low mental health resilience, whereas at high levels of responsivity, boys' mental health resilience was similar to girls. The association between mental health and behaviour and parental responsivity is likely bidirectional. Boys showed lower mental health resilience and their behaviour and mental health problems may differ in nature from girls, which may influence parents' responsivity. However, parenting behaviour did not differ between boys and girls in either cohort. This suggests that preterm‐born boys are particularly vulnerable to the adverse effects of low levels of responsivity on their mental health.

In addition to parenting practices directed at the child – second layer of Figure 1 – Morris, Silk, Steinberg, Myers, and Robinson's (2007) tripartite model sheds light on how family relationships – third layer of Figure 1 – impact children's adjustment directly and indirectly through emotion regulation. Observations of adults' emotional displays and interactions and the family's emotional climate play an important role (Morris et al., 2007). A key component of this emotional climate is the interparental relationship. We consistently found that interparental relationship quality was positively related to mental health resilience in preterm‐born children. Although the literature mainly focused on interparental conflict, a recent meta‐analysis showed that relationship quality was positively related to children's psychosocial outcomes to the same extent as conflict (Van Eldik et al., 2020). In BLS, interparental relationship quality was more strongly associated with mental health resilience in preterm boys than girls. This contrasts meta‐analytic findings showing stronger associations between interparental conflict and emotionality/internalising problems in non‐preterm girls (Van Eldik et al., 2020).

The peer group – layer four of Figure 1 – also influences children's resilience. We found consistent evidence of the detrimental impact of bullying, including both victimisation (peers) and perpetration (peers, siblings). Preterm‐born children are more often victimised by peers than term‐born children (Wolke, Baumann, et al., 2015). A proactive approach to bullying is therefore warranted, addressing victimisation and perpetration in both peer and family contexts, as sibling bullying is known to carry over to peer bullying (Wolke, Tippett, & Dantchev, 2015). Moreover, family factors, including adversity, domestic violence, child maltreatment, inconsistent/harsh parenting, lack of warmth and monitoring, and interparental conflict, are associated with bullying victimisation and perpetration in peer and sibling contexts (Dantchev & Wolke, 2019; Nocentini, Fiorentini, Di Paola, & Menesini, 2019; Wolke, Tippett, & Dantchev, 2015), whereas parental involvement, support, and open communication protect against peer victimisation (Nocentini et al., 2019). Bullying should be assessed using parent, teacher, and child reports, as parents are not always aware of children's involvement (Harcourt, Jasperse, & Green, 2014). Knowing that preterm‐born children are more often bullied, follow‐up assessments should routinely inquire about bullying.

At the most distal level – outer layer of Figure 1 – child‐friendly and higher‐quality neighbourhoods were associated with increased mental health resilience in preterm‐born children in the UK cohort, also after accounting for family socioeconomic characteristics. This may in part be driven by access to green space and playgrounds (Flouri, Midouhas, & Joshi, 2014). In families with low levels of adversity, parents' feelings of safety in their neighbourhood were positively associated with mental health resilience in preterm‐born children. However, when family adversity was high, greater feelings of neighbourhood safety were unexpectedly associated with lower mental health resilience. Neighbourhood safety may not sufficiently buffer against the stronger effects of family adversity, whereas quality of the physical home environment – a more proximal context – was positively associated with resilience when family adversity was high. The negative association between safety and mental health resilience may reflect an increasing discrepancy between the child's experiences inside and outside the home.

The effects of promotive factors were generally similar, but their prevalence varied largely based on contextual adversity, particularly in the MCS. In half of the cases, these differences were subsequently related to resilience. In BLS, contextual adversity had less impact on the degree of protective/promotive factors and on mental health resilience via these factors. This suggests that other mechanisms, such as educational and social policies, might mitigate the negative impact of contextual adversity on mental health resilience. Differences in conceptualisation between cohorts hamper further study of such mechanisms, but the differences in findings show that while contextual adversity may be considered inevitable, its consequences for child development are not fixed. Alternatively, despite similar domains of contextual adversity, differences in the measurements of adversities across samples may explain the differing findings. Perhaps adversities measured in the MCS are more relevant or severe, resulting in a stronger association between contextual adversity and protective/promotive factors and mental health resilience.

### Practical implications

Factors that moderated the association between sex or contextual adversity and resilience are considered *protective* factors. They had a particularly strong effect on resilience in preterm‐born children facing higher risks of mental health problems (i.e. boys and those exposed to contextual adversity). These protective factors are potential targets for prevention/intervention for these specific risk groups. Factors that increased resilience irrespective of sex and contextual adversity (i.e. main effects) are considered *promotive* factors as they promote resilience in all preterm‐born children. These factors are relevant for the development of general prevention, interventions, and policies aimed at improving outcomes following preterm birth. Factors that mediated the association between sex or contextual adversity and resilience are considered *mitigating* factors if they had a positive impact and *exacerbating* factors if they had a negative impact on resilience. Depending on the direction of the relation, interventions could increase or decrease these factors specifically in boys or children facing contextual adversity, thereby potentially increasing resilience in these groups.

Figure 3 shows candidate factors for each of these purposes that could be used to inform a multilayered approach fostering mental health resilience in preterm‐born children. The first layer could involve universal support, encompassing the promotion of factors beneficial to all preterm‐born children. Levers for which we found consistent evidence include improving self‐regulation, cognitive abilities, parent–child relationship, and the interparental relationship, an authoritative and structured family climate, and reducing bullying. The second layer could entail additional targeted support for specific risk groups. Based on our findings, preterm boys may benefit from interventions improving (cognitive) self‐regulation, parental responsivity, interparental relationship, and sibling bullying. Negative consequences of contextual adversity could be mitigated by interventions targeting emotional dysregulation, parental responsivity, mother–child conflict, interparental relationship quality, sibling relationship quality, and bullying, the physical home environment, and neighbourhood quality. Timely identification of difficulties in factors associated with mental health resilience (Figure 3) could guide personalised support tailored to the unique needs of each child, thereby potentially optimising mental health outcomes. Currently, follow‐up assessments of preterm‐born children focus on individual child outcomes. Our findings stress the importance of the multiple systems the child is embedded in. Therefore, we advocate for follow‐up of the family as a whole instead of the individual preterm child, starting in the hospital and continuing after discharge.

### Limitations

We used a comprehensive approach to assessing protective, promotive, and risk factors across multisystem levels, considering differential effects of sex and contextual adversity to understand what could foster mental health resilience in preterm‐born children. Despite variation in conceptualisations, the consistency of findings across cohorts and regions enhances the generalisability of our results and their potential to inform policies. Racial/ethnic groups were not considered, warranting further investigation. Contextual adversity was measured before protective/promotive factors, which were assessed before outcomes, ensuring temporal precedence. However, measuring resilience at one timepoint disregarded its dynamic nature. Quantification approaches of childhood adversity have been criticised for their narrow range of adversities, neglecting the important impact of economic hardship (Tzouvara et al., 2023). Our measure included adverse life events, family adversity, psychosocial distress, and socioeconomic deprivation. However, cumulative approaches obscure the effects and interplay of single adversities. Despite our efforts to account for this, selective drop‐out may have affected the degree of resilience and protective/promotive factors, but associations between them are less likely to be affected by characteristics associated with drop‐out (Wolke et al., 2009), as confirmed by the limited number of interactions in our study. The interrelatedness of factors was considered, but most mediation/moderation analyses were based on single‐factor models. The actual relations between protective/promotive factors, sex, contextual adversity, and resilience are presumably more complex, warranting further research. Whereas the present study focused on general mental health, future studies could also further differentiate between internalising and externalising behaviour to potentially identify differential protective/promotive factors. Lastly, relying on single informants, primarily mothers, limited our consideration of variation in behaviour across contexts and the influence of informants' characteristics and their relationship with the child.

## Conclusion

Modifiable protective, promotive, and risk factors across multiple systems collectively explain a substantial portion of variation in mental health resilience among preterm‐born children. This underscores the high potential for improving mental health outcomes after preterm birth. By shifting focus on resilience, we illuminated avenues for targeted prevention and intervention with a dual focus on enhancing protective/promotive factors while mitigating risks. Regulatory abilities, interparental relationship, and bullying seem particularly promising primary targets. The emphasis on resilience rather than deficits has the added benefit of reducing the risk of stigmatisation among at‐risk groups. Moreover, it is more appealing to work towards positive goals. Nonetheless, our findings stress the importance of addressing specific needs of vulnerable groups. Moving forward, a comprehensive approach recognising the interplay of multisystem factors is essential for improving mental health outcomes in preterm‐born children.

## Ethical considerations

The BLS was ethically approved by the Ethics Committee of the University of Munich Children's Hospital and the Bavarian Health Council (Landesärztekammer). All parents provided informed consent. For the MCS, ethical approval was obtained from the Multi‐centre Research Ethics Committees of London (3 and 5 years) and Yorkshire (7 years). Written consent was required from parents or guardians.

**Key points**

- Children born preterm are at increased risk of mental health problems and their mental health outcomes have not improved in the past decades.
- This is the first comprehensive analysis of a wide range of modifiable factors that could potentially promote mental health resilience in preterm‐born children.
- The findings suggest that there is high potential for improving mental health outcomes of preterm‐born children and that this may be achieved through a variety of levers for intervention and prevention across all multisystem levels.
- Regulatory abilities, interparental relationship quality, and bullying seem particularly promising levers.
- The evidence underscores the importance of the larger context in which development takes place. Follow‐up after preterm birth should therefore focus on the family as a whole instead of the individual child, starting in the hospital and continuing after discharge.

## Supporting information


**Figure S1**. Participant flowchart of the Bavarian Longitudinal Study (A) and the Millennium Cohort Study (B).
**Table S1**. Overview of the instruments and variables used to measure each construct.
Appendix S1
**Table S2**. Descriptive statistics for protective, promotive, and risk factors and proportion of missing values for the Bavarian Longitudinal Study (*n*=574) and Millennium Cohort Study (*n*=985) samples.
Appendix S2
**Figure S2**. Unstandardised interaction effects of sex (A, B) and contextual adversity (C–E) with protective factors on mental health resilience in the Bavarian Longitudinal Study (A) and the Millennium Cohort Study (B–E).
**Table S3**. Unstandardised test statistics of interaction terms for each construct with sex and contextual adversity in the Bavarian Longitudinal Study.
**Table S4**. Unstandardised test statistics of interaction terms for each construct with sex and contextual adversity in the Millennium Cohort Study.
**Table S5**. Regression models of the association between individual‐level constructs and mental health resilience adjusted for prenatal tobacco exposure in the Bavarian Longitudinal Study and prenatal tobacco and alcohol exposure in the Millennium Cohort Study.
**Figure S3**. Differences in mental health resilience and protective, promotive, and risk factors according to sex (A, C) and contextual adversity (B, D) in the Bavarian Longitudinal Study (A, B) and the Millennium Cohort Study (C, D).
**Table S6**. Indirect effects of sex and contextual adversity on mental health resilience through protective, promotive and risk factors in the Bavarian Longitudinal Study.
**Table S7**. Indirect effects of sex and contextual adversity on mental health resilience through protective, promotive and risk factors in the Millennium Cohort Study.

## Data Availability

Data from the Bavarian Longitudinal Study are available after access request through the RECAP Preterm Data Platform (https://platform.recap‐preterm.eu/). Data from the Millennium Cohort Study are available through the UK Data Service (https://ukdataservice.ac.uk/).
