# Supplementary material for: Fostering positive mental health outcomes in vulnerable children: Pathways to resilience after preterm birth
Source: J Child Psychol Psychiatry. 2025 Jul 16;66(12):1860–74. doi: 10.1111/jcpp.70002 (PMC12626177; doi:10.1111/jcpp.70002)
Supplement: Supplementary file 1 — Figure S1. Participant flowchart of the Bavarian Longitudinal Study (A) and the Millennium Cohort Study (B). Table S1. Overview of the instruments and variables used to measure each construct. Appendix S1 Table S2. Descriptive statistics for protective, promotive, and risk factors and proportion of missing values for the Bavarian Longitudinal Study (n=574) and Millennium Cohort Study (n=985) samples. Appendix S2 Figure S2. Unstandardised interaction effects of sex (A, B) and contextual adversity (C–E) with protective factors on mental health resilience in the Bavarian Longitudinal Study (A) and the Millennium Cohort Study (B–E). Table S3. Unstandardised test statistics of interaction terms for each construct with sex and contextual adversity in the Bavarian Longitudinal Study. Table S4. Unstandardised test statistics of interaction terms for each construct with sex and contextual adversity in the Millennium Cohort Study. Table S5. Regression models of the association between individual‐level constructs and mental health resilience adjusted for prenatal tobacco exposure in the Bavarian Longitudinal Study and prenatal tobacco and alcohol exposure in the Millennium Cohort Study. Figure S3. Differences in mental health resilience and protective, promotive, and risk factors according to sex (A, C) and contextual adversity (B, D) in the Bavarian Longitudinal Study (A, B) and the Millennium Cohort Study (C, D). Table S6. Indirect effects of sex and contextual adversity on mental health resilience through protective, promotive and risk factors in the Bavarian Longitudinal Study. Table S7. Indirect effects of sex and contextual adversity on mental health resilience through protective, promotive and risk factors in the Millennium Cohort Study. [file JCPP-66-1860-s001.docx]

**Table of contents**

**Figure S1**

Participant flowchart of the Bavarian Longitudinal Study (A) and the Millennium Cohort Study (B) **2**

**Table S1**

Overview of the instruments and variables used to measure each construct **4**

**Appendix S1** **7**

**Table S2**

Descriptive statistics for protective, promotive, and risk factors and proportion of missing values for

the Bavarian Longitudinal Study (*n*=574) and Millennium Cohort Study (*n*=985) samples. **10**

**Appendix S2** **12**

**Figure S2**

Unstandardised interaction effects of sex (A-B) and contextual adversity (C-E) with protective factors on mental health resilience in the Bavarian Longitudinal Study (A) and the Millennium Cohort Study (B-E). **13**

**Table S3**

Unstandardised test statistics of interaction terms for each construct with sex and contextual adversity in the Bavarian

Longitudinal Study **14**

**Table S4**

Unstandardised test statistics of interaction terms for each construct with sex and contextual adversity in the Millennium

Cohort Study **16**

**Table S5**

Regression models of the association between individual-level constructs and mental health resilience

adjusted for prenatal tobacco exposure in the Bavarian Longitudinal Study and prenatal tobacco and alcohol

exposure in the Millennium Cohort Study. **17**

**Figure S3**

Differences in mental health resilience and protective, promotive, and risk factors according to sex (A, C)

and contextual adversity (B, D) in the Bavarian Longitudinal Study (A, B) and the Millennium Cohort

Study (C, D). **18**

**Table S6**

Indirect effects of sex and contextual adversity on mental health resilience through protective, promotive,

and risk factors in the Bavarian Longitudinal Study **22**

**Table S7**

Indirect effects of sex and contextual adversity on mental health resilience through protective, promotive,

and risk factors in the Millennium Cohort Study **23**

**Figure S1. Participant flowchart of the Bavarian Longitudinal Study (A) and the Millennium Cohort Study (B).**

*Note.* GA = gestational age, MCS = Millennium Cohort Study, SES = socioeconomic status.


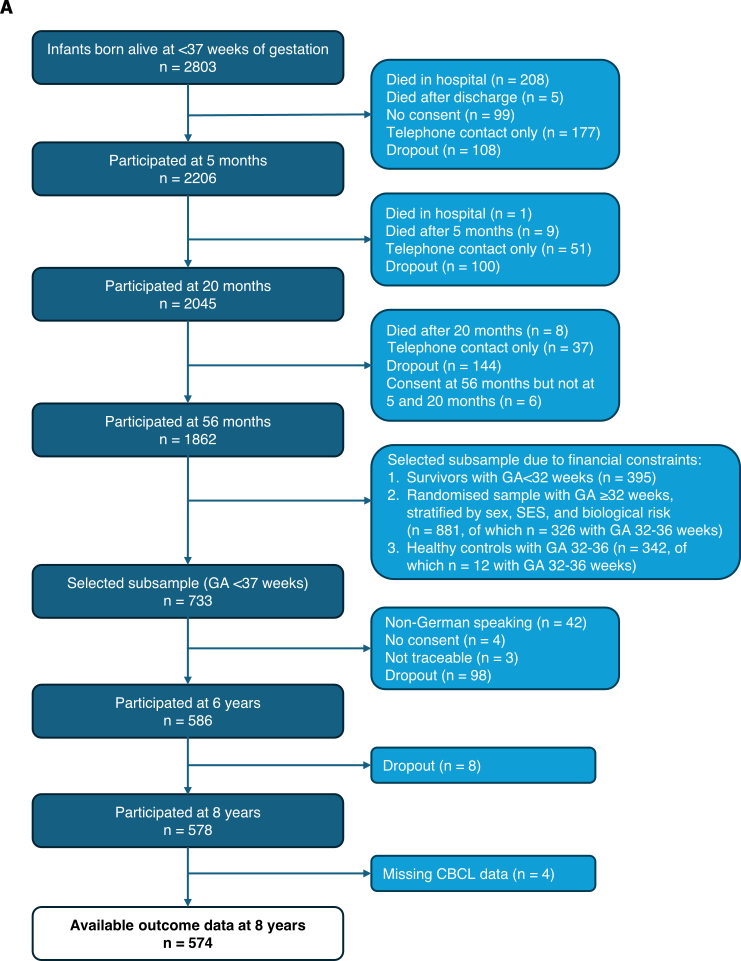


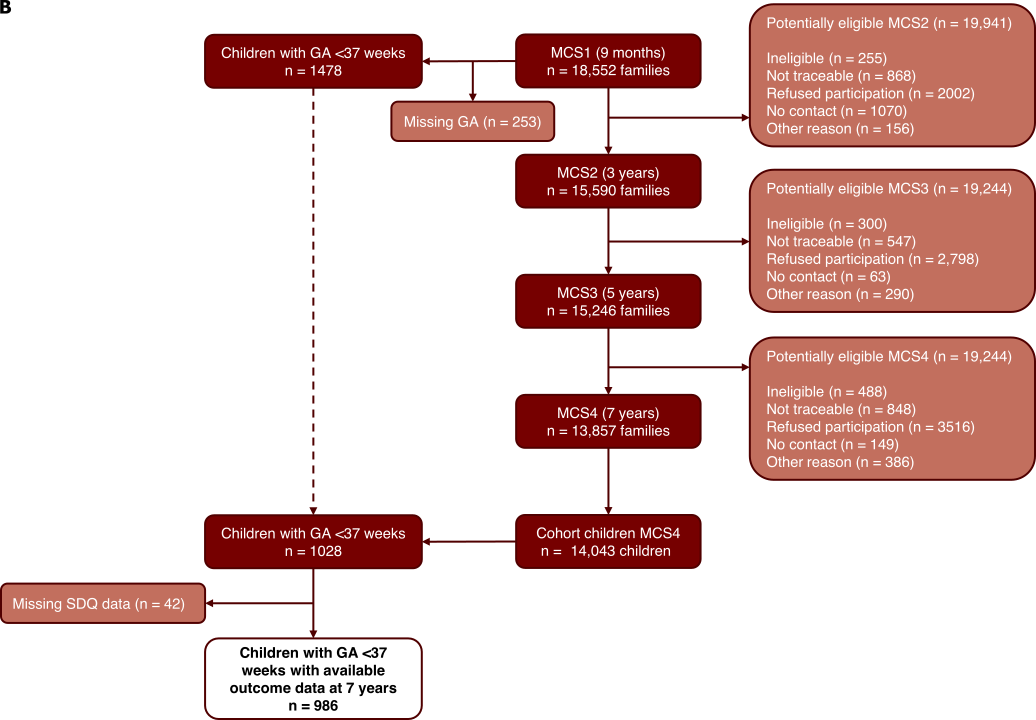


**Table S1. Overview of the instruments and variables used to measure each construct.**

| **Construct** | **Instrument** | **Indicators or subscales** | **Variable type** | **Reliability** | | **CFA fit** | | | | **Factor**  **loading** |
| --- | --- | --- | --- | --- | --- | --- | --- | --- | --- | --- |
|  |  |  |  | ω | *H* | Chi^2^ *p* | CFI | RMSEA | SRMR |  |
| **Bavarian Longitudinal Study** | | | | | | | | | | |
| Self-regulation/ temperament | Emotionality, Activity, Sociability (EAS) – 6y | Activity | Multidimensional construct; observed continuous subscale scores | 0.71 |  |  |  |  |  |  |
|  |  | Effortful control |  | 0.81 |  |  |  |  |  |  |
|  |  | Shyness |  | 0.91 |  |  |  |  |  |  |
|  |  | Sociability |  | 0.74 |  |  |  |  |  |  |
|  |  | Emotionality |  | 0.72 |  |  |  |  |  |  |
| Social-emotional regulation | Tester’s Rating of Child Behaviour (TRCB) – 6y | Approach/withdrawal | Latent construct (CFA) with ordinal indicators (1-9) |  | 0.95 | 0.465 | 1.00 | 0.00 | 0.01 | 0.86 |
|  |  | Adaptability |  |  |  |  |  |  |  | 0.90 |
|  |  | General emotional tone |  |  |  |  |  |  |  | 0.79 |
|  |  | Social attractiveness |  |  |  |  |  |  |  | 0.81 |
| Attention | Team Rating of Child Attention (TEAM) – 6y | Sum score of Demandingness, Attention, Robustness and endurance | Observed continuous | 0.88 |  |  |  |  |  |  |
| Parent-perceived competence | Harter Scale (parent-rated) – 6y | Cognition | Multidimensional construct; observed continuous subscale scores | 0.75 |  |  |  |  |  |  |
|  |  | Peer relationships |  | 0.79 |  |  |  |  |  |  |
|  |  | Motor skills |  | 0.81 |  |  |  |  |  |  |
|  |  | Maternal relationship |  | 0.60 |  |  |  |  |  |  |
| Cognitive abilities | Kaufman Assessment Battery for Children (KABC) – 6y | Total IQ | Observed continuous | NA |  |  |  |  |  |  |
| Social inhibition | Social Inhibition Assessment | Composite reaction time by averaging verbal and non-verbal reaction time (Spearman ρ = 0.66) | Observed continuous; data were transformed to normalise distribution^a^ | NA |  |  |  |  |  |  |
| Sensitivity | Assessment of Mother-Child-Interactions with the Etch-a-Sketch (AMCIES) – 6y | Verbal control | Latent construct (CFA) with ordinal indicators (1-9 or 1-5) |  | 0.81 | 0.166 | 0.99 | 0.04 | 0.02 | 0.50 |
|  |  | Criticism |  |  |  |  |  |  |  | 0.91 |
|  |  | Non-verbal control |  |  |  |  |  |  |  | 0.45 |
|  |  | Harmony |  |  |  |  |  |  |  | 0.79 |
| Mother-child relationship | Friendship and Family Interview, Card-sorting task – 6y | Affection | Latent construct (CFA) with ordinal indicators (-2 to +2) |  | 0.70 | 0.008 | 0.99 | 0.06 | 0.04 | 0.59 |
|  |  | Care |  |  |  |  |  |  |  | 0.68 |
|  |  | Comfort |  |  |  |  |  |  |  | 0.72 |
|  |  | Cuddling |  |  |  |  |  |  |  | 0.50 |
|  |  | Conflict |  |  |  |  |  |  |  | 0.42 |
| Father-child relationship | Friendship and Family Interview, Card-sorting task – 6y | Affection | Latent construct (CFA) with ordinal indicators (-2 to +2) |  | 0.62 | 0.092 | 0.99 | 0.04 | 0.03 | 0.61 |
|  |  | Care |  |  |  |  |  |  |  | 0.55 |
|  |  | Comfort |  |  |  |  |  |  |  | 0.64 |
|  |  | Cuddling |  |  |  |  |  |  |  | 0.45 |
|  |  | Bad conscience/trust |  |  |  |  |  |  |  | 0.45 |
| Sibling relationships | Friendship and Family Interview, Card-sorting task – 6y | Fun/Fooling around | Latent construct (CFA) with ordinal indicators (-2 to +2) |  | 0.75 | 0.007 | 0.99 | 0.05 | 0.03 | 0.56 |
|  |  | Playing |  |  |  |  |  |  |  | 0.73 |
|  |  | Prosocial behaviour |  |  |  |  |  |  |  | 0.69 |
|  |  | Cuddling |  |  |  |  |  |  |  | 0.51 |
|  |  | Conflict |  |  |  |  |  |  |  | 0.49 |
|  |  | Affection |  |  |  |  |  |  |  | 0.68 |
| Home learning environment | Home Observation for Measurement of the Environment (HOME) – 6y | Tried to teach names animals | Latent construct (CFA) with dichotomous indicators (0-1) |  | 0.76 | 0.001 | 0.98 | 0.06 | 0.08 | 0.75 |
|  |  | Tried to teach alphabet |  |  |  |  |  |  |  | 0.61 |
|  |  | Tried to teach colours |  |  |  |  |  |  |  | 0.84 |
|  |  | Tried to teach shapes |  |  |  |  |  |  |  | 0.64 |
|  |  | Tried to teach numbers |  |  |  |  |  |  |  | 0.85 |
|  |  | Tried to teach spatial relations |  |  |  |  |  |  |  | 0.76 |
| Family climate | Family Environment Scale (FES) – 6y | Positive climate, sum of: | Observed continuous |  |  |  |  |  |  |  |
|  |  | Cohesion |  | 0.66 |  |  |  |  |  |  |
|  |  | Propensity for conflict |  | 0.78 |  |  |  |  |  |  |
|  |  | Normative-authoritative climate, sum: |  |  |  |  |  |  |  |  |
|  |  | Control |  | 0.81 |  |  |  |  |  |  |
|  |  | Organisation |  | 0.71 |  |  |  |  |  |  |
|  |  | Moral-religious emphasis |  | 0.77 |  |  |  |  |  |  |
|  |  | Stimulating climate, sum: |  |  |  |  |  |  |  |  |
|  |  | Active-recreational orientation |  | 0.70 |  |  |  |  |  |  |
|  |  | Intellectual-cultural orientation |  | 0.82 |  |  |  |  |  |  |
| Interparental relationship | Dyadic Adjustment Scale (DAS) – 6y | Total DAS score | Observed continuous | 0.96 |  |  |  |  |  |  |
| Social support | Parent Interview: Frequency of social contacts – 6y | Sum score: | Observed continuous | 0.71 |  |  |  |  |  |  |
|  |  | Visits from friends, relatives, neighbours |  |  |  |  |  |  |  |  |
|  |  | Visiting friends, relatives, neighbours |  |  |  |  |  |  |  |  |
|  |  | Calls from friends, relatives, neighbours |  |  |  |  |  |  |  |  |
|  |  | Calling friends, relatives, neighbours to chat |  |  |  |  |  |  |  |  |
| Bullying | Parent Interview – 6y | Victimisation, sum of: | Observed dichotomous (0-1) | NA |  |  |  |  |  |  |
|  |  | Other children irritate, insult, bully |  |  |  |  |  |  |  |  |
|  |  | Child gets beaten by others |  |  |  |  |  |  |  |  |
|  |  | Perpetration, sum of: |  | NA |  |  |  |  |  |  |
|  |  | Child irritates, insults, bullies others |  |  |  |  |  |  |  |  |
|  |  | Child fights with others |  |  |  |  |  |  |  |  |
| Friendship quality | Friendship and Family Interview, Card-sorting task – 6y | Fun/Fooling around | Latent construct (CFA) with ordinal indicators (-2 to +2) |  | 0.80 | 0.128 | 0.99 | 0.03 | 0.03 | 0.45 |
|  |  | Playing |  |  |  |  |  |  |  | 0.74 |
|  |  | Prosocial behaviour |  |  |  |  |  |  |  | 0.76 |
|  |  | Cuddling |  |  |  |  |  |  |  | 0.47 |
|  |  | Conflict |  |  |  |  |  |  |  | 0.61 |
|  |  | Affection |  |  |  |  |  |  |  | 0.72 |
| Child-friendliness neighbourhood | Living Situation Questionnaire – 6y | How child-friendly do you find your neighbourhood? | Observed ordinal (0-3) | NA |  |  |  |  |  |  |
| **Millennium Cohort Study** | | | | | | | | | | |
| Self-regulation | Child Social Behaviour Questionnaire (CSBQ) – 5y | Independence and self-regulation | Observed; continuous | NA |  |  |  |  |  |  |
|  |  | Emotional dysregulation |  | NA |  |  |  |  |  |  |
| Cognitive abilities | British Ability Scales (BAS) – 5y | Naming Vocabulary | Observed; continuous | NA |  |  |  |  |  |  |
|  |  | Picture Similarities |  | NA |  |  |  |  |  |  |
|  |  | Pattern Construction |  | NA |  |  |  |  |  |  |
| Emotional & verbal responsivity | HOME – 3y | Positive voice when speaking to child | Latent construct (CFA); dichotomous indicators |  | 0.72 | 0.59 | 1.00 | 0.00 | 0.02 | 0.92 |
|  |  | Converses at least twice with child |  |  |  |  |  |  |  | 0.76 |
|  |  | Praises child spontaneously |  |  |  |  |  |  |  | 0.83 |
|  |  | Caresses or kisses child |  |  |  |  |  |  |  | 0.89 |
| Parental warmth | Child-Parent Relationship Scale (CPRS) – 3y | Closeness | Observed; continuous | NA |  |  |  |  |  |  |
|  |  | Conflict |  | NA |  |  |  |  |  |  |
| Maternal attachment | Condon questionnaire – 9 months | Feelings of annoyance/ irritation | Latent construct (CFA); ordinal indicators (1-4 or 1-3) |  | 0.60 | 0.14 | 1.00 | 0.03 | 0.02 | 0.61 |
|  |  | Feelings when caring for baby |  |  |  |  |  |  |  | 0.64 |
|  |  | Feelings of patience when with baby |  |  |  |  |  |  |  | 0.64 |
|  |  | Feelings about giving up things due to baby |  |  |  |  |  |  |  | 0.49 |
| Home learning environment | HOME – 3y | Anyone at home helps or tries to teach the child: | Latent construct (CFA); ordinal indicators (0-7) |  | 0.68 | 0.01 | 0.99 | 0.05 | 0.04 |  |
|  |  | Reading |  |  |  |  |  |  |  | 0.36 |
|  |  | Painting |  |  |  |  |  |  |  | 0.40 |
|  |  | Alphabet |  |  |  |  |  |  |  | 0.62 |
|  |  | Numbers |  |  |  |  |  |  |  | 0.77 |
|  |  | Songs |  |  |  |  |  |  |  | 0.57 |
| Physical home environment | HOME – 3y | In-home play environment is safe | Latent construct (CFA); dichotomous indicators |  | 0.88 | 0.62 | 1.00 | 0.00 | 0.02 | 0.87 |
|  |  | Home interior is dark |  |  |  |  |  |  |  | 0.74 |
|  |  | House is reasonably clean |  |  |  |  |  |  |  | 0.99 |
|  |  | House is reasonably uncluttered |  |  |  |  |  |  |  | 0.88 |
| Interparental relationship | Golombok Rust Inventory of Marital State – 5y | Partner is sensitive to and aware of my needs | Latent construct (CFA); ordinal indicators (1-5, 1-8, or 1-6) |  | 0.87 | 0.00 | 1.00 | 0.06 | 0.03 | 0.80 |
|  |  | Partner doesn't listen to me |  |  |  |  |  |  |  | 0.83 |
|  |  | I sometimes feel lonely even with partner |  |  |  |  |  |  |  | 0.80 |
|  |  | I am likely to separate from partner |  |  |  |  |  |  |  | 0.79 |
|  |  | How often do you and partner disagree over issues relating to child? |  |  |  |  |  |  |  | 0.41 |
|  |  | How happy are you with relationship with partner? |  |  |  |  |  |  |  | 0.68 |
| Parents' social support | Single questionnaire items – 3 and 5y | Nobody to share feelings with (3y) | Observed, ordinal (1-5) | NA |  |  |  |  |  |  |
|  |  | Family would help if financial problems (3y) |  | NA |  |  |  |  |  |  |
|  |  | Frequency of seeing friends (5y) |  | NA |  |  |  |  |  |  |
| Family routines | Single questionnaire items – 5y | Regular bedtimes | Observed; ordinal (1-4) | NA |  |  |  |  |  |  |
|  |  | Regular mealtimes |  | NA |  |  |  |  |  |  |
| Sibling relationship | Single questionnaire items – 5y | Likes to be with sibling | Sum score of average across siblings; observed, continuous | 0.63 |  |  |  |  |  |  |
|  |  | Not much interested in sibling |  |  |  |  |  |  |  |  |
|  |  | Has a lot of fun with sibling |  |  |  |  |  |  |  |  |
|  |  | Teases or needles sibling | Single item averaged across siblings; observed continuous | NA |  |  |  |  |  |  |
| Friendships | Single questionnaire item – 5y | How often does child spend time with friends? | Observed, ordinal (1-6) | NA |  |  |  |  |  |  |
| Observed neighbourhood quality | Observer ratings – 3y | General condition of residences/buildings | Latent construct (CFA); ordinal indicators (1-4, 1-3, or 1-2) |  | 0.88 | 0.00 | 0.99 | 0.05 | 0.09 | 0.84 |
|  |  | Security blinds, gates, bars |  |  |  |  |  |  |  | 0.66 |
|  |  | Rubbish/litter in the street |  |  |  |  |  |  |  | 0.83 |
|  |  | Dog mess on the pavement |  |  |  |  |  |  |  | 0.67 |
|  |  | Graffiti |  |  |  |  |  |  |  | 0.85 |
|  |  | Evidence of vandalism |  |  |  |  |  |  |  | 0.84 |
|  |  | Arguing, fighting or hostile behaviour |  |  |  |  |  |  |  | 0.42 |
|  |  | How did you feel? |  |  |  |  |  |  |  | 0.90 |
|  |  | Abandoned cars |  |  |  |  |  |  |  | 0.46 |
| Perceived safety and child friendliness | Single questionnaire items – 5y | Good area to bring up a child? | Observed, ordinal (1-5) | NA |  |  |  |  |  |  |
|  |  | How safe do you feel? |  | NA |  |  |  |  |  |  |

*Note.* CFA = confirmatory factor analysis; NA = not applicable due to single or few items or not available because item-level data were not available to compute composite reliability.

^a^ ordered quantile normalization; Peterson & Cavanaugh (2019)

**Appendix S1**

*Contextual adversity*

In the BLS, contextual adversity was defined as the unweighted sum of psychosocial stress, family adversity, and socioeconomic deprivation. Ordered quantile normalisation (Peterson & Cavanaugh, 2019) was applied to normalise the distribution of the sum score. Psychosocial stress was measured with the Psychosocial Stress Index (PSI) (Riegel et al., 1995) in the neonatal period and at 5, 20, and 56 months. The index was based on 14 items, related to physical and mental health problems of parents, financial difficulties, death of family members, excessive work pressure, serious conflicts, and social isolation. Presence of these stressors were scored as 1, resulting in an index score with a minimum score of 0 and a maximum score of 14 at each of the four time points. Family adversity was measured at the same time points with the Family Adversity Index (FAI) (Riegel et al., 1995), based on 8 items related to early parenthood, overcrowded living space, parents without educational qualification, single parenthood, large number of children, infant requiring home or external care, lack of social support, and parental psychiatric problems. Presence of these adversities were scored as 1, resulting in an index score with a minimum score of 0 and a maximum score of 8 at each of the four time points. Socioeconomic deprivation was defined as a monthly family income <25^th^ percentile reported at 6 years of age. The specific items are listed in the table below.

| **Psychosocial Stress Index (PSI)** | **Family Adversity Index (FAI)** | **Socioeconomic deprivation** |
| --- | --- | --- |
| 1. Recent death of a family member, relative or close friend | 1. Early parenthood (<20 years of age) | Monthly family income after tax and social insurance contributions <25^th^ percentile (i.e. <3000 Deutsche Mark) |
| 1. Excess pressure from work on the father (husband/partner) | 1. Overcrowded living space (<50 m^2^ per person) |  |
| 1. Financial difficulties | 1. Parents without educational qualification or vocational training |  |
| 1. Behavioural or health problems of a sibling | 1. Single parenthood (single, divorced, or widowed) or a very tense partnership between parents |  |
| 1. Marital or partnership problems | 1. Large number of children in the family (≥4 children) |  |
| 1. Physical health problems/serious illness or exhaustion of the mother | 1. Infant requiring home or external care (e.g. foster care) |  |
| 1. Mental health problems or psychiatric disorder of the mother | 1. No social support available in emergency situations |  |
| 1. Excess pressure from work on the mother | 1. Parental psychiatric problems (disclosed by parent or suspected by interviewer). |  |
| 1. Physical health problems/serious illness or exhaustion of the father |  |  |
| 1. Mental health problems or psychiatric disorder of the father |  |  |
| 1. Social isolation |  |  |
| 1. Mother has no time for her own interests |  |  |
| 1. Father has no time for his own interests |  |  |
| 1. Serious conflicts with family or friends |  |  |

In the MCS, contextual adversity was defined as the unweighted sum of psychosocial distress, adverse life events, and socioeconomic deprivation across the three previous follow-up assessments at 9 months, 3 years, and 5 years. Psychosocial distress was measured with the Rutter Malaise Inventory at 9 months (ω=0.77), with a cut-off of ≥4 indicating distress. At 3 and 5 years, the Kessler 6 was used with a cut-off of ≥5 indicating distress (Prochaska et al., 2012). Adverse life events included the following experiences or events reported at 9 months, 3 years, or 5 years: domestic violence, verbal and/or physical maltreatment, high alcohol consumption, regular drug use, divorce, death of parent, and being placed in foster care. Each event was counted once, resulting in a minimum score of 0 and a maximum score of 8. Socioeconomic deprivation was measured at the area and family level. Area deprivation was defined as the lowest decile of the Index of Multiple Deprivation (IMD) in any of the previous sweeps. Family level deprivation was defined as <60% of the median poverty indicator on the OECD equivalence scale in any of the previous sweeps.

*GAM*

Generalised additive models (GAM) were fitted with *K*=10 spline basis functions, determining the complexity or “wiggliness” of the curve. To prevent overfitting, the wiggliness is constrained by the smoothing parameter, which was selected by restricted marginal likelihood. To determine whether *K* was large enough, it was verified that the effective degrees of freedom (EDF) for each term were well below *K*-1. The model’s Akaike Information Criterion (AIC) was corrected for uncertainty in smoothing parameter estimation (Wood et al., 2016).

*Potential bias in full-regression model coefficients*

Full regression models including all observed and latent protective/promotive factors, adjusted for sex and contextual adversity, were used to explore independent effects of factors on mental health resilience. Such complex models are prone to misspecification and convergence problems (Devlieger et al., 2019). Regression with factor scores have therefore been proposed but these are known to produce biased regression coefficients. A solution has recently been offered by the local structural-after-measurement (SAM) approach (Rosseel & Loh, 2022), using the *sam()* function in *lavaan.* With SAM, the measurement part of the model is estimated before the structural part. Options for missing data within this framework are still under development, limiting the usability of this approach for the current study. However, we applied both solutions in a complete case analysis to get an understanding of the degree of bias of factor score regression and the impact on the results.

The results of both approaches are presented in the tables below. Comparison of SAM and factor score regression in complete cases (BLS: *n*=421, MCS: *n*=245) resulted in similar conclusions regarding the effects of latent constructs in terms of significance. The standardised regression coefficients were highly similar in the MCS but varied slightly across models in the BLS.

**References**

Devlieger, I., Talloen, W., & Rosseel, Y. (2019). New developments in factor score regression: Fit indices and a model comparison test. *Educ Psychol Meas*, *79*(6), 1017-1037.

Peterson, R. A., & Cavanaugh, J. E. (2019). Ordered quantile normalization: a semiparametric transformation built for the cross-validation era. *J Appl Stat*, *47*(13-15), 2312–2327.

Prochaska, J. J., Sung, H. Y., Max, W., Shi, Y., & Ong, M. (2012). Validity study of the K6 scale as a measure of moderate mental distress based on mental health treatment need and utilization. *Int J Methods Psychiatr Res*, *21*(2), 88-97.

Riegel, K., Ohrt, B., Wolke, D., & Österlund, K. (1995). *Die Entwicklung gefährdet geborener kinder bis zum fünften lebensjahr. [The development of children born at risk until their fifth year of life]* (F. E. Verlag, Ed.).

Rosseel, Y., & Loh, W. W. (2022). A structural after measurement approach to structural equation modeling. *Psychol Methods*.

Wood, S. N., Pya, N., & Säfken, B. (2016). Smoothing parameter and model selection for general smooth models. *J Am Stat Assoc*, *111*(516), 1548-1563.

**Bavarian Longitudinal Study: comparison of regression models using a local structural-after-measurement (SAM) approach and factor scores for latent constructs in a full model with complete cases (*n* = 421).**

| SAM approach | | | | | Regression with factor scores | | | | |
| --- | --- | --- | --- | --- | --- | --- | --- | --- | --- |
|  | *b* | SE | *p* | β |  | *b* | SE | *p* | β |
| Effortful control | -0.45 | 0.15 | 0.003 | -0.15 | Effortful control | -0.44 | 0.15 | 0.003 | -0.15 |
| Activity | 0.20 | 0.17 | 0.251 | 0.06 | Activity | 0.20 | 0.17 | 0.228 | 0.06 |
| Shyness | -0.02 | 0.07 | 0.806 | -0.01 | Shyness | -0.01 | 0.07 | 0.899 | -0.01 |
| Emotionality | 0.57 | 0.14 | 0.000 | 0.20 | Emotionality | 0.55 | 0.14 | 0.000 | 0.19 |
| Sociability | -0.14 | 0.18 | 0.441 | -0.05 | Sociability | -0.12 | 0.18 | 0.491 | -0.04 |
| Social-emotional regulation, *latent variable* | -0.64 | 0.49 | 0.185 | **-0.06** | Social-emotional regulation, *factor score* | -0.87 | 0.84 | 0.297 | **-0.05** |
| Attention | -0.05 | 0.15 | 0.759 | -0.02 | Attention | -0.06 | 0.15 | 0.712 | -0.02 |
| Cognition (parent-perceived) | 0.43 | 1.57 | 0.786 | 0.02 | Cognition (parent-perceived) | 0.39 | 1.60 | 0.805 | 0.01 |
| Peers (parent-perceived) | -1.67 | 1.47 | 0.258 | -0.06 | Peers (parent-perceived) | -1.98 | 1.47 | 0.178 | -0.07 |
| Motor (parent-perceived) | -1.45 | 1.00 | 0.147 | -0.08 | Motor (parent-perceived) | -1.42 | 1.00 | 0.157 | -0.07 |
| Maternal relationship (parent-perceived) | -1.67 | 1.53 | 0.275 | -0.06 | Maternal relationship (parent-perceived) | -1.59 | 1.52 | 0.294 | -0.06 |
| Cognitive abilities | 0.08 | 0.06 | 0.193 | 0.08 | Cognitive abilities | 0.07 | 0.06 | 0.241 | 0.07 |
| Social inhibition | -0.42 | 0.60 | 0.485 | -0.03 | Social inhibition | -0.41 | 0.61 | 0.502 | -0.03 |
| Maternal sensitivity, *latent variable* | -0.34 | 0.86 | 0.691 | **-0.02** | Maternal sensitivity, *factor score* | -1.90 | 1.42 | 0.182 | **-0.06** |
| Mother-child relationship, *latent variable* | -7.14 | 2.69 | 0.008 | **-0.21** | Mother-child relationship, *factor score* | -4.25 | 1.21 | 0.000 | **-0.17** |
| Father-child relationship, *latent variable* | -5.44 | 2.34 | 0.020 | **-0.18** | Father-child relationship, *factor score* | -3.17 | 1.13 | 0.005 | **-0.13** |
| Sibling relationships, *latent variable* | -1.54 | 2.44 | 0.529 | **-0.05** | Sibling relationships, *factor score* | 0.35 | 1.25 | 0.782 | **0.01** |
| Home learning environment, *latent variable* | -1.07 | 4.47 | 0.811 | **-0.01** | Home learning environment, *factor score* | -0.91 | 1.14 | 0.426 | **-0.04** |
| Positive family climate | -0.01 | 0.08 | 0.934 | 0.00 | Positive family climate | -0.01 | 0.08 | 0.931 | 0.00 |
| Normative-authoritative | -0.12 | 0.05 | 0.022 | -0.10 | Normative-authoritative | -0.11 | 0.05 | 0.041 | -0.09 |
| Stimulating family climate | 0.11 | 0.05 | 0.039 | 0.11 | Stimulating family climate | 0.09 | 0.05 | 0.088 | 0.09 |
| Interparental relationship | -0.10 | 0.03 | 0.005 | -0.13 | Interparental relationship | -0.09 | 0.03 | 0.006 | -0.13 |
| Parents' frequency of social contacts | 0.03 | 0.17 | 0.867 | 0.01 | Parents' frequency of social contacts | 0.03 | 0.17 | 0.879 | 0.01 |
| Bullying perpetration | -0.75 | 1.40 | 0.594 | -0.03 | Bullying perpetration | -0.68 | 1.39 | 0.624 | -0.03 |
| Bullying victimisation | 3.37 | 1.29 | 0.009 | 0.13 | Bullying victimisation | 3.11 | 1.29 | 0.016 | 0.12 |
| Peer relationships, *latent variable* | -3.65 | 2.26 | 0.106 | **-0.13** | Peer relationships, *factor score* | -2.29 | 1.58 | 0.148 | **-0.08** |
| Child-friendliness neighbourhood | -0.22 | 0.76 | 0.772 | -0.01 | Child-friendliness neighbourhood | -0.21 | 0.75 | 0.780 | -0.01 |

*Note.* Standardised coefficients (β) of latent constructs are shown in bold for ease of comparison.

**Millennium Cohort Study: comparison of regression models using a local structural-after-measurement (SAM) approach and factor scores for latent constructs in a full model with complete cases (*n* = 245).**

| SAM approach | | | | | Regression with factor scores | | | | |
| --- | --- | --- | --- | --- | --- | --- | --- | --- | --- |
|  | *b* | SE | *p* | β |  | *b* | SE | *p* | β |
| Independence and self-regulation | -0.94 | 0.81 | 0.245 | -0.06 | Independence and self-regulation | -0.87 | 0.81 | 0.284 | -0.06 |
| Emotional dysregulation | 4.15 | 0.69 | 0.000 | 0.39 | Emotional dysregulation | 4.19 | 0.71 | 0.000 | 0.39 |
| Closeness subscale | -0.09 | 0.11 | 0.424 | -0.05 | Closeness subscale | -0.11 | 0.11 | 0.347 | -0.05 |
| Conflict subscale | 0.16 | 0.06 | 0.006 | 0.18 | Conflict subscale | 0.14 | 0.06 | 0.020 | 0.15 |
| Naming Vocabulary | 0.00 | 0.03 | 0.982 | 0.00 | Naming Vocabulary | 0.00 | 0.03 | 0.888 | -0.01 |
| Picture Similarities | 0.00 | 0.03 | 0.967 | 0.00 | Picture Similarities | 0.00 | 0.03 | 0.994 | 0.00 |
| Pattern Construction | -0.03 | 0.03 | 0.359 | -0.05 | Pattern Construction | -0.03 | 0.03 | 0.300 | -0.06 |
| Theory of Mind | -0.29 | 0.71 | 0.684 | -0.02 | Theory of Mind | -0.29 | 0.72 | 0.684 | -0.02 |
| Maternal attachment, *latent variable* | 0.57 | 1.22 | 0.640 | **0.03** | Maternal attachment, *factor score* | -0.15 | 0.74 | 0.838 | **-0.01** |
| Emotional and verbal responsivity, *latent variable* | 2.25 | 4.47 | 0.615 | **0.04** | Emotional and verbal responsivity, *factor score* | 0.46 | 0.63 | 0.470 | **0.04** |
| Physical home environment, *latent variable* | -6.40 | 3.81 | 0.092 | **-0.11** | Physical home environment, *factor score* | -1.54 | 0.75 | 0.039 | **-0.11** |
| Home learning environment, *latent variable* | 0.45 | 1.28 | 0.726 | **0.02** | Home learning environment, *factor score* | 0.31 | 0.94 | 0.743 | **0.02** |
| Sibling relationship | -0.12 | 0.25 | 0.619 | -0.03 | Sibling relationship | -0.16 | 0.25 | 0.540 | -0.03 |
| Teases or needles sibling | 0.14 | 0.43 | 0.747 | -0.02 | Teases or needles sibling | 0.15 | 0.44 | 0.735 | -0.02 |
| Regular bedtimes | 0.30 | 0.34 | 0.387 | 0.05 | Regular bedtimes | 0.34 | 0.34 | 0.321 | 0.05 |
| Regular mealtimes | 0.00 | 0.50 | 0.996 | 0.00 | Regular mealtimes | -0.07 | 0.50 | 0.897 | -0.01 |
| Interparental relationship, *latent variable* | -0.39 | 0.34 | 0.249 | **-0.06** | Interparental relationship, *factor score* | -0.33 | 0.41 | 0.428 | **-0.05** |
| Nobody to share feelings with | 0.17 | 0.31 | 0.584 | 0.03 | Nobody to share feelings with | 0.17 | 0.32 | 0.601 | 0.03 |
| Family would help if financial problems | -0.03 | 0.26 | 0.914 | -0.01 | Family would help if financial problems | -0.03 | 0.26 | 0.901 | -0.01 |
| Frequency of seeing friends | -0.03 | 0.33 | 0.916 | -0.01 | Frequency of seeing friends | 0.04 | 0.33 | 0.914 | 0.01 |
| How often child spends time with friends | 0.01 | 0.21 | 0.964 | 0.00 | How often child spends time with friends | 0.03 | 0.21 | 0.882 | 0.01 |
| Neighbourhood quality, *latent variable* | -1.29 | 0.57 | 0.025 | **-0.13** | Neighbourhood quality, *factor score* | -0.87 | 0.44 | 0.045 | **-0.13** |
| Good area to bring up child | -0.68 | 0.41 | 0.098 | -0.12 | Good area to bring up child | -0.62 | 0.44 | 0.157 | -0.10 |
| Feeling safe | 0.82 | 0.45 | 0.072 | 0.12 | Feeling safe | 0.73 | 0.46 | 0.115 | 0.11 |

*Note.* Standardised coefficients (β) of latent constructs are shown in bold for ease of comparison.

**Table S2. Descriptive statistics for protective, promotive, and risk factors and proportion of missing values for the Bavarian Longitudinal Study (*n*=574) and Millennium Cohort Study (*n*=985) samples.**

| **Construct** | **Instrument** | **Variable** | **Weighted M (SD) [range] or %** | **Missing**  **n (%)** |
| --- | --- | --- | --- | --- |
| **Bavarian Longitudinal Study** | | | | |
| Self-regulation/ temperament | Emotionality, Activity, Sociability (EAS) | Activity | 18.78 (3.92) [5.00, 25.00] | 39 (7%) |
|  |  | Effortful control | 16.63 (4.36) [5.00, 25.00] |  |
|  |  | Shyness | 31.64 (10.70) [13.00, 63.00] |  |
|  |  | Sociability | 22.49 (4.44) [9.00, 30.00] |  |
|  |  | Emotionality | 16.36 (4.33) [6.00, 30.00] |  |
| Social-emotional regulation | Tester’s Rating of Child Behaviour (TRCB) | Latent variable |  | 39 (7%) |
| Attention | Team Rating of Child Attention (TEAM) | Sum score | 16.50 (5.43) [3.00, 27.00] | 34 (6%) |
| Parent-perceived competence | Harter Scale (parent-rated) | Cognition | 2.81 (0.53) [1.00, 3.83] | 21 (4%) |
|  |  | Peer relationships | 2.25 (0.48) [0.83, 3.50] |  |
|  |  | Motor skills | 2.82 (0.71) [0.50, 4.00] |  |
|  |  | Maternal relationship | 2.95 (0.43) [0.67, 4.00] |  |
| Cognitive abilities | Kaufman Assessment Battery for Children (KABC) | Total IQ | 90.66 (15.47) [43.00, 124.00] | 33 (6%) |
| Social inhibition | Social Inhibition Assessment | Average of verbal and non-verbal reaction time | -0.02 (1.06) [-2.72, 3.38] | 63 (11%) |
| Sensitivity | Assessment of Mother-Child-Interactions with the Etch-a-Sketch (AMCIES) | Latent variable |  | 44 (8%) |
|  |  |  |  |  |
| Mother-child relationship | Friendship and Family Interview, Card-sorting task | Latent variable |  | 32 (6%) |
| Father-child relationship | Friendship and Family Interview, Card-sorting task | Latent variable |  | 32 (6%) |
| Sibling relationships | Friendship and Family Interview, Card-sorting task | Latent variable |  | 32 (6%) |
| Home learning environment | Home Observation for Measurement of the Environment (HOME) | Latent variable |  | 32 (6%) |
| Family climate | Family Environment Scale (FES) | Positive climate | 99.86 (6.51) [81.03, 118.49] | 40 (7%) |
|  |  | Normative-authoritative climate | 99.86 (10.76) [67.38, 128.36] | 39 (7%) |
|  |  | Stimulating climate | 99.47 (12.88) [62.49, 120.46] | 39 (7%) |
| Interparental relationship | Dyadic Adjustment Scale (DAS) | Total DAS score | 133.75 (17.04) [34.00, 169.00] | 64 (11%) |
| Social support | Parent Interview: Frequency of social contacts | Sum score | 13.50 (3.54) [4.00, 20.00] | 33 (6%) |
| Bullying | Parent Interview | Victimisation | 34.7% | 33 (6%) |
|  |  | Perpetration | 26.6% | 32 (6%) |
| Friendship quality | Friendship and Family Interview, Card-sorting task | Latent variable |  | 32 (6%) |
| Child friendliness neighbourhood | Living Situation Questionnaire | How child-friendly do you find your neighbourhood? | Not child friendly: 1.4%  A little child friendly: 10.8%  Rather child friendly: 38.4%  Very child friendly: 43.1% | 36 (6%) |
| Covariates | Biological sex at birth | Male sex | 52.7% | 0 (0%) |
|  | Psychosocial Stress Index (PSI), Family Adversity Index (FAI), Socioeconomic deprivation (single questionnaire item) | Contextual adversity (untransformed) | 5.96 (4.98) [0.00, 32.00] | 0 (0%) |
| **Millennium Cohort Study** | | | | |
| Self-regulation | Child Social Behaviour Questionnaire (CSBQ) | Independence and self-regulation | 2.48 (0.36) [1.00, 3.00] | 59 (6%) |
|  |  | Emotional dysregulation | 1.77 (0.47) [1.00, 3.00] |  |
| Cognitive abilities | British Ability Scales (BAS) | Naming Vocabulary | 53.66 (11.69) [20.00, 80.00] | 46 (5%) |
|  |  | Picture Similarities | 54.02 (11.30) [20.00, 80.00] |  |
|  |  | Pattern Construction | 48.06 (10.91) [20.00, 80.00] |  |
| Emotional & verbal responsivity | HOME | Latent variable |  | 163 (17%) |
| Parental warmth | Child-Parent Relationship Scale (CPRS) | Closeness | 33.37 (2.57) [7.00, 35.00] | 239 (24%) |
|  |  | Conflict | 17.57 (6.20) [8.00, 38.00] | 208 (21%) |
| Maternal attachment | Condon questionnaire | Latent variable |  | 29 (3%) |
| Home learning environment | HOME | Latent variable |  | 99 (10%) |
| Physical home environment | HOME | Latent variable |  | 164 (17%) |
| Interparental relationship | Golombok Rust Inventory of Marital State | Latent variable |  | 273 (28%) |
| Parents' social support | Single questionnaire items | Nobody to share feelings with (3y) | Strongly agree: 1.9%  Agree: 4.9%  Neither agree nor disagree: 8.5%  Disagree: 29.5%  Strongly disagree: 25.8% | 304 (31%) |
|  |  | Family would help if financial problems (3y) | Strongly agree: 33.2%  Agree: 22.9%  Neither agree nor disagree: 6.7%  Disagree: 3.2%  Strongly disagree: 3.2% | 313 (32%) |
|  |  | Frequency of seeing friends (5y) | Every day: 10.3%  3-6 times: 17.9%  1-2 times: 47.0%  Not at all: 19.8%  No friends: 0.9% | 48 (5%) |
| Family routines | Single questionnaire items | Regular bedtimes | (almost) Never: 5.9%  Sometimes: 3.6%  Usually: 26.1%  Always: 60.3% | 48 (5%) |
|  |  | Regular mealtimes | (almost) Never: 3.5%  Sometimes: 3.5%  Usually: 33.2%  Always: 55.7% |  |
| Sibling relationship | Single questionnaire items | Likes to be with sibling | 8.07 (1.12) [4.00, 9.00] | 345 (35%) |
|  |  | Not much interested in sibling |  |  |
|  |  | Has a lot of fun with sibling |  |  |
|  |  | Teases or needles sibling | 1.91 (0.64) [1.00, 3.00] |  |
| Peer relationships | Single questionnaire item | How often does child spend time with friends? | (almost) Everyday: 10.2%  Several times a week: 16.5%  Once or twice a week: 30.5%  Once or twice a month: 15.6%  Less often: 10.7%  Not at all: 12.4% | 48 (5%) |
| Observed neighbourhood quality | Observer ratings | Latent variable |  | 119 (12%) |
| Perceived safety and child friendliness | Single questionnaire items | Good area to bring up child? | Very poor: 3.2%  Poor: 5.0%  Average: 19.3%  Good: 40.4%  Excellent: 27.8% | 49 (5%) |
|  |  | How safe do you feel? | Very unsafe: 1.3%  Fairly unsafe: 4.8%  Neither safe nor unsafe: 9.1%  Fairly safe: 50.9%  Very safe: 29.7% | 48 (5%) |
| Covariates | Biological sex at birth | Male sex | 54.4% | 0 (0%) |
|  | Rutter Malaise Inventory, Kessler 6, adverse life events (single questionnaire items), and socioeconomic deprivation (single questionnaire items) | Contextual adversity | 2.19 (1.64) [0.00, 8.00] | 0 (0%) |

**Appendix S2**

To define resilience, gestational age and birthweight were used as indicators of prematurity. The reasons additional neonatal factors were not included in the model are threefold: 1. The literature is inconclusive regarding additional neonatal factors that may further increase the risk for mental health problems (Linsell et al., 2018); 2. As a general population-based cohort, information on neonatal morbidity is lacking for the MCS preterm sample and would only have been available for the BLS sample; 3. Most neonatal factors and morbidities are correlated with gestational age and birth weight. By including these characteristics, the measure of mental health resilience accounts sufficiently for neonatal risk exposure.

Nevertheless, it was explored whether the selected model including gestational age only (with a non-linear smooth term) would be improved by additional inclusion of other neonatal risk factors: small for gestational age (SGA), intraventricular haemorrhage (IVH), bronchopulmonary dysplasia (BPD), and postnatal sepsis (PNS). This exploratory analysis was only done in the BLS sample, because these data were not available for the MCS sample. As can be seen below, the other neonatal factors were not associated with mental health outcomes at 8 years (CBCL total score) and inclusion of these factors in addition to gestational age did not improve model fit, as indicated by the increased AIC.

Model 1 - selected model as described in manuscript: Y ~ s(GA)

Model 2 - model with additional neonatal factor: Y ~ s(GA) + SGA + IVH + BPD + PNS

**Model estimates of model 2:**

|  | *EDF** |  | *p* |
| --- | --- | --- | --- |
| GA (smooth term) | 2.435 |  | **<0.001** |
|  | *estimate* | *SE* | *p* |
| SGA | 1.660 | 1.804 | 0.358 |
| IVH | 0.056 | 0.825 | 0.946 |
| BPD | 0.994 | 1.679 | 0.554 |
| PNS | 0.118 | 1.568 | 0.940 |

*An EDF>1 indicates that the relation is non-linear.

**Model comparison:**

|  | ANOVA | | | | | corrected AIC | |
| --- | --- | --- | --- | --- | --- | --- | --- |
|  | Resid. df | Resid. dev | df | deviance | *p* | df | AIC |
| Model 1 | 339.31 | 99938 |  |  |  | 5.012 | **2808.453** |
| Model 2 | 335.37 | 99610 | 3.9361 | 328.05 | 0.8874 | 8.973 | 2815.246 |

**References**

Linsell, L., Malouf, R., Johnson, S., Morris, J., Kurinczuk, J. J., & Marlow, N. (2016). Prognostic factors for behavioral

problems and psychiatric disorders in children born very preterm or very low birth weight: a systematic review. *Journal of Developmental & Behavioral Pediatrics*, *37*(1), 88-102.

**Figure S2. Unstandardised interaction effects of sex (A-B) and contextual adversity (C-E) with protective factors on mental health resilience in the Bavarian Longitudinal Study (A) and the Millennium Cohort Study (B-E).**

**
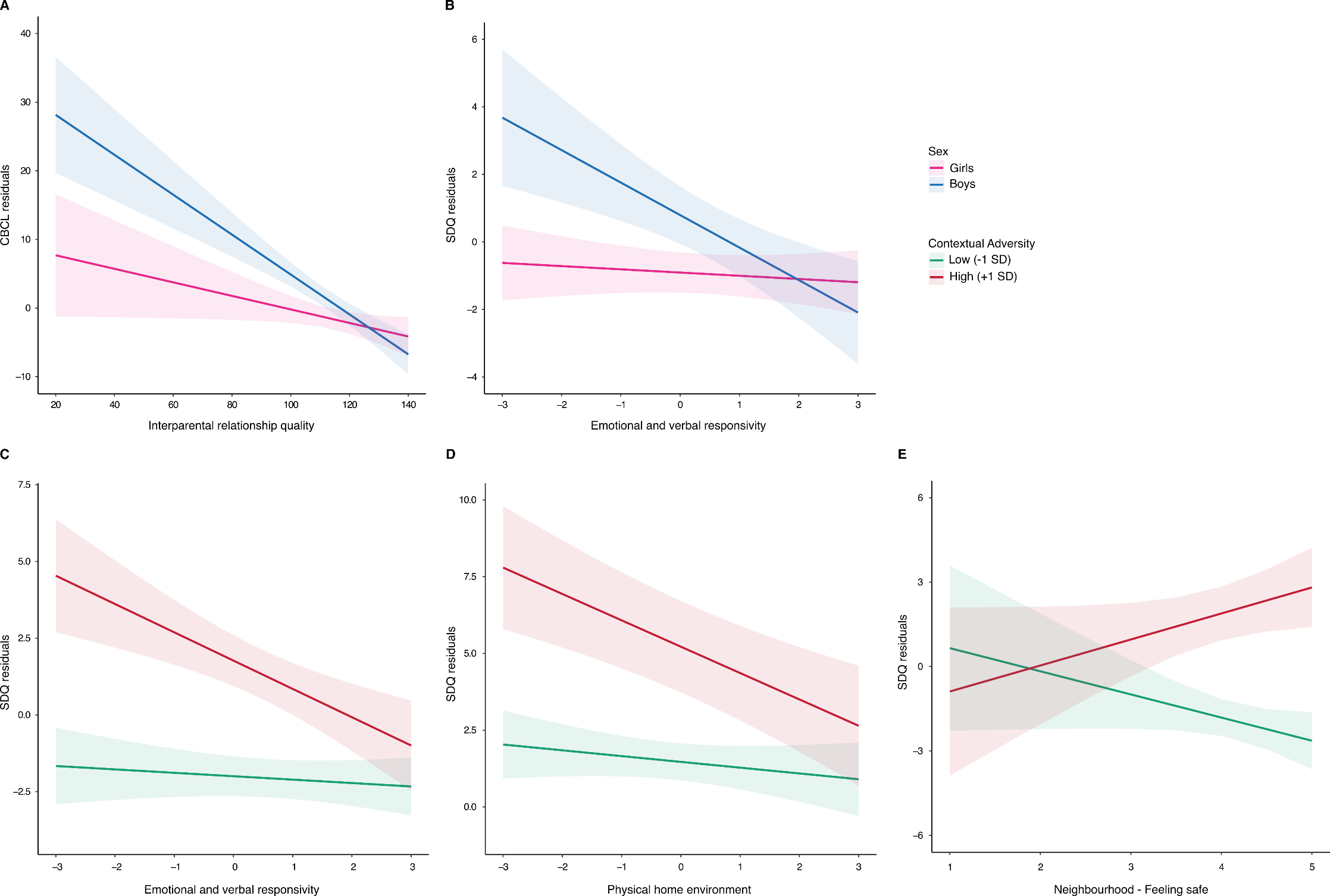
**

**Table S3. Unstandardised test statistics of interaction terms for each construct with sex and contextual adversity in the Bavarian Longitudinal Study.**

| System level | Instrument | Subscale/item | estimate | SE | lower | upper | *p* | FDR-corrected *p* |
| --- | --- | --- | --- | --- | --- | --- | --- | --- |
| Individual | Self-regulation/temperament (EAS) | Effortful control × sex | -0.360 | 0.351 | -1.049 | 0.329 | 0.306 | 0.471 |
|  |  | Effortful control × context | -0.056 | 0.215 | -0.477 | 0.365 | 0.795 | 0.758 |
|  |  | Activity × sex | 0.597 | 0.351 | -0.092 | 1.285 | 0.089 | 0.471 |
|  |  | Activity × context | -0.043 | 0.146 | -0.329 | 0.242 | 0.766 | 0.758 |
|  |  | Shyness × sex | 0.098 | 0.146 | -0.188 | 0.385 | 0.501 | 0.605 |
|  |  | Shyness × context | -0.089 | 0.082 | -0.250 | 0.071 | 0.276 | 0.443 |
|  |  | Emotionality × sex | -0.278 | 0.316 | -0.898 | 0.341 | 0.379 | 0.522 |
|  |  | Emotionality × context | -0.027 | 0.164 | -0.348 | 0.294 | 0.868 | 0.785 |
|  |  | Sociability × sex | -0.766 | 0.379 | -1.509 | -0.023 | 0.043 | 0.135 |
|  |  | Sociability × context | -0.261 | 0.186 | -0.625 | 0.103 | 0.160 | 0.315 |
|  | Tester’s Rating of Child Behaviour | Social-emotional regulation × sex^a^ | 0.261 | 0.438 | -0.598 | 1.120 | 0.551 | 0.635 |
|  |  | Social-emotional regulation × context | -0.084 | 0.217 | -0.509 | 0.341 | 0.698 | 0.726 |
|  | Team Rating of Child Attention | Attention × sex | -0.368 | 0.264 | -0.886 | 0.150 | 0.164 | 0.320 |
|  |  | Attention × context | -0.171 | 0.121 | -0.409 | 0.067 | 0.158 | 0.313 |
|  | Perceived competence (Harter Scales), parent-rated | Cognition × sex | -2.672 | 2.967 | -8.486 | 3.143 | 0.368 | 0.513 |
|  |  | Cognition × context | 0.969 | 1.321 | -1.620 | 3.559 | 0.463 | 0.575 |
|  |  | Peers × sex | 0.165 | 3.033 | -5.778 | 6.109 | 0.957 | 0.842 |
|  |  | Peers × context | 0.582 | 1.418 | -2.198 | 3.362 | 0.682 | 0.718 |
|  |  | Motor × sex | 2.796 | 2.094 | -1.308 | 6.899 | 0.182 | 0.340 |
|  |  | Motor × context | -1.561 | 1.123 | -3.762 | 0.640 | 0.165 | 0.320 |
|  |  | Maternal relationship × sex | -0.857 | 3.243 | -7.213 | 5.499 | 0.792 | 0.758 |
|  |  | Maternal relationship × context | 0.460 | 1.800 | -3.069 | 3.989 | 0.798 | 0.758 |
|  | Kaufman Assessment Battery for Children (KABC) | Cognitive abilities × sex | -0.084 | 0.093 | -0.267 | 0.099 | 0.368 | 0.513 |
|  |  | Cognitive abilities × context | -0.073 | 0.044 | -0.158 | 0.013 | 0.095 | 0.230 |
|  | Social Inhibition Assessment | Social inhibition × sex | -0.443 | 1.437 | -3.260 | 2.374 | 0.758 | 0.750 |
|  |  | Social inhibition × context | -1.446 | 0.711 | -2.839 | -0.052 | 0.042 | 0.135 |
| Parent-child | AMCIES | Maternal sensitivity × sex^b^ | -2.646 | 1.602 | -5.787 | 0.494 | 0.099 | 0.236 |
|  |  | Maternal sensitivity × context | -0.042 | 0.721 | -1.455 | 1.371 | 0.954 | 0.841 |
|  | Card-Sorting Task | Mother-child relationship × sex^a^ | -0.037 | 0.050 | -3.162 | 1.460 | 0.467 | 0.579 |
|  |  | Mother-child relationship × context | -0.001 | 0.045 | -1.004 | 0.980 | 0.981 | 0.850 |
|  |  | Father-child relationship × sex^c^ | 2.078 | 1.176 | -0.228 | 4.384 | 0.077 | 0.205 |
|  |  | Father-child relationship × context | -0.948 | 0.580 | -2.085 | 0.189 | 0.102 | 0.240 |
|  |  | Sibling relationship × sex^b^ | 0.177 | 1.157 | -2.091 | 2.444 | 0.879 | 0.791 |
|  |  | Sibling relationship × context | 0.404 | 0.514 | -0.603 | 1.411 | 0.431 | 0.555 |
| Family | Home Observation for Measurement of the Environment (HOME) | Home learning environment × sex^b^ | 0.483 | 1.154 | -1.779 | 2.744 | 0.676 | 0.718 |
|  |  | Home learning environment × context | -1.023 | 0.526 | -2.054 | 0.008 | 0.052 | 0.149 |
|  | Family climate (FES) | Positive family climate × sex | -0.008 | 0.203 | -0.406 | 0.390 | 0.968 | 0.846 |
|  |  | Positive family climate × context | -0.135 | 0.112 | -0.355 | 0.085 | 0.229 | 0.396 |
|  |  | Normative-authoritative × sex | 0.119 | 0.123 | -0.122 | 0.360 | 0.333 | 0.495 |
|  |  | Normative-authoritative × context | -0.151 | 0.069 | -0.285 | -0.016 | 0.028 | 0.100 |
|  |  | Stimulating family climate × sex | -0.020 | 0.099 | -0.215 | 0.175 | 0.841 | 0.781 |
|  |  | Stimulating family climate × context | -0.026 | 0.049 | -0.122 | 0.069 | 0.591 | 0.662 |
|  | Dyadic Adjustment Scale (DAS) | Interparental relationship × sex | -0.225 | 0.077 | -0.376 | -0.074 | 0.004 | **0.021** |
|  |  | Interparental relationship × context | 0.045 | 0.041 | -0.036 | 0.125 | 0.277 | 0.443 |
|  | Parent interview | Parents' frequency of social contacts × sex | 0.119 | 0.387 | -0.641 | 0.878 | 0.760 | 0.750 |
|  |  | Parents' frequency of social contacts × context | 0.278 | 0.181 | -0.076 | 0.632 | 0.124 | 0.265 |
| Peer group | Bullying | Perpetration × sex | -0.122 | 3.124 | -6.245 | 6.000 | 0.969 | 0.846 |
|  |  | Perpetration × context | -2.216 | 1.545 | -5.245 | 0.813 | 0.152 | 0.305 |
|  |  | Victimisation × sex | 0.946 | 2.659 | -4.265 | 6.158 | 0.722 | 0.734 |
|  |  | Victimisation × context | 0.488 | 1.265 | -1.992 | 2.968 | 0.700 | 0.726 |
|  | Card-Sorting Task | Peer relationships × sex^b^ | -3.599 | 1.841 | -7.208 | 0.010 | 0.051 | 0.147 |
|  |  | Peer relationships × context | -0.167 | 0.904 | -1.938 | 1.604 | 0.854 | 0.783 |
| Neighbourhood | Parent interview | Child-friendliness neighbourhood × sex | -0.637 | 1.980 | -4.518 | 3.243 | 0.748 | 0.750 |
|  |  | Child-friendliness neighbourhood × context | -1.434 | 1.072 | -3.534 | 0.666 | 0.181 | 0.340 |

*Note.* AMCIES = Assessment of Mother-Child-Interactions with the Etch-a-Sketch, FDR = false discovery rate, SE = standard error.

^a^ Metric invariance could not be tested because there were too few cases in one of the categories in one of the sexes.

^b^ Metric invariance was supported across sex.

^c^ Metric invariance was not supported; factor loadings differed across sex (χ^2^(4) = 15.96, *p*=0.003).

**Table S4. Unstandardised test statistics of interaction terms for each construct with sex and contextual adversity in the Millennium Cohort Study.**

| System level | Instrument | Subscale/item | estimate | SE | lower | upper | *p* | FDR-corrected *p* |
| --- | --- | --- | --- | --- | --- | --- | --- | --- |
| Individual | Child Social Behaviour Questionnaire (CSBQ) | Independence and self-regulation × sex | -1.713 | 1.368 | -4.395 | 0.968 | 0.210 | 0.371 |
|  |  | Independence and self-regulation × context | 0.075 | 0.425 | -0.757 | 0.907 | 0.860 | 0.785 |
|  |  | Emotional dysregulation × sex | 2.938 | 1.239 | 0.509 | 5.367 | 0.018 | 0.071 |
|  |  | Emotional dysregulation × context | 0.747 | 0.324 | 0.112 | 1.381 | 0.021 | 0.082 |
|  | British Ability Scales (BAS) | Naming Vocabulary × sex | -0.093 | 0.047 | -0.185 | -0.002 | 0.046 | 0.140 |
|  |  | Picture Similarities × sex | -0.015 | 0.056 | -0.125 | 0.094 | 0.781 | 0.758 |
|  |  | Pattern Construction × sex | 0.026 | 0.057 | -0.085 | 0.138 | 0.645 | 0.708 |
|  |  | Naming Vocabulary × context | -0.007 | 0.014 | -0.034 | 0.019 | 0.585 | 0.658 |
|  |  | Picture Similarities × context | 0.018 | 0.019 | -0.018 | 0.055 | 0.329 | 0.492 |
|  |  | Pattern Construction × context | -0.039 | 0.020 | -0.078 | 0.000 | 0.053 | 0.150 |
| Parent-child | Home Observation for Measurement of the Environment (HOME) | Emotional & verbal responsivity × sex^a^ | -0.203 | 0.064 | -0.328 | -0.077 | 0.002 | **0.012** |
|  |  | Emotional & verbal responsivity × context | -0.191 | 0.061 | -0.311 | -0.070 | 0.002 | **0.012** |
|  | Child-Parent Relationship Scale (CPRS) | Closeness subscale × sex | 0.135 | 0.297 | -0.447 | 0.717 | 0.650 | 0.710 |
|  |  | Closeness subscale × context | 0.001 | 0.099 | -0.194 | 0.196 | 0.989 | 0.851 |
|  |  | Conflict subscale × sex | 0.062 | 0.129 | -0.191 | 0.315 | 0.631 | 0.700 |
|  |  | Conflict subscale × context | -0.020 | 0.032 | -0.082 | 0.042 | 0.531 | 0.623 |
|  | Condon questionnaire | Maternal attachment × sex^a^ | -0.071 | 0.065 | -0.197 | 0.056 | 0.274 | 0.443 |
|  |  | Maternal attachment × context | -0.038 | 0.059 | -0.154 | 0.078 | 0.521 | 0.619 |
| Family | HOME | Home learning environment × sex^a^ | -0.036 | 0.061 | -0.154 | 0.083 | 0.557 | 0.638 |
|  |  | Home learning environment × context | -0.127 | 0.072 | -0.268 | 0.014 | 0.078 | 0.205 |
|  | HOME | Physical home environment × sex^a^ | -0.107 | 0.064 | -0.232 | 0.018 | 0.092 | 0.226 |
|  |  | Physical home environment × context | -0.141 | 0.054 | -0.247 | -0.035 | 0.009 | **0.041** |
|  | Golombok Rust Inventory of Marital State | Interparental relationship quality × sex^a^ | 0.009 | 0.105 | -0.197 | 0.215 | 0.930 | 0.823 |
|  |  | Interparental relationship quality × context | 0.047 | 0.113 | -0.175 | 0.270 | 0.678 | 0.718 |
|  | Parents' social support, parent interview | Frequency of seeing friends × sex | -0.161 | 0.607 | -1.351 | 1.030 | 0.791 | 0.758 |
|  |  | Frequency of seeing friends × context | -0.324 | 0.183 | -0.684 | 0.035 | 0.077 | 0.205 |
|  |  | Nobody to share feelings with × sex | -0.572 | 0.685 | -1.915 | 0.772 | 0.404 | 0.540 |
|  |  | Nobody to share feelings with × context | 0.319 | 0.184 | -0.041 | 0.679 | 0.083 | 0.212 |
|  |  | Family would help if financial problems × sex | -0.008 | 0.731 | -1.441 | 1.424 | 0.991 | 0.851 |
|  |  | Family would help if financial problems × context | -0.007 | 0.193 | -0.386 | 0.372 | 0.972 | 0.847 |
|  | Family routines, parent interview | Regular bedtimes × sex | -0.084 | 0.907 | -1.862 | 1.694 | 0.926 | 0.823 |
|  |  | Regular bedtimes × context | 0.350 | 0.271 | -0.182 | 0.881 | 0.198 | 0.353 |
|  |  | Regular mealtimes × sex | 0.083 | 0.966 | -1.811 | 1.977 | 0.931 | 0.823 |
|  |  | Regular mealtimes × context | 0.045 | 0.256 | -0.458 | 0.547 | 0.861 | 0.785 |
|  | Sibling relationship, parent interview | Sibling relationship quality × sex | -0.659 | 0.562 | -1.761 | 0.443 | 0.241 | 0.405 |
|  |  | Sibling relationship quality × context | -0.263 | 0.169 | -0.595 | 0.068 | 0.119 | 0.259 |
|  |  | Teases or needles sibling × sex | 0.190 | 0.973 | -1.716 | 2.096 | 0.845 | 0.781 |
|  |  | Teases or needles sibling × context | 0.084 | 0.328 | -0.558 | 0.726 | 0.797 | 0.758 |
| Peer group | Friendships, parent interview | How often child spends time with friends × sex | 0.003 | 0.364 | -0.711 | 0.717 | 0.994 | 0.851 |
|  |  | How often child spends time with friends × context | 0.114 | 0.107 | -0.096 | 0.324 | 0.288 | 0.451 |
| Neighbourhood | Tester’s observation | Observed neighbourhood quality × sex^b^ | -0.085 | 0.045 | -0.174 | 0.003 | 0.059 | 0.164 |
|  |  | Observed neighbourhood quality × context | -0.026 | 0.042 | -0.109 | 0.056 | 0.534 | 0.624 |
|  | Parent interview | Good area to bring up child × sex | -0.380 | 0.587 | -1.531 | 0.771 | 0.517 | 0.617 |
|  |  | Good area to bring up child × context | -0.155 | 0.194 | -0.535 | 0.225 | 0.424 | 0.555 |
|  |  | Feeling safe × sex | 0.275 | 0.710 | -1.118 | 1.667 | 0.699 | 0.726 |
|  |  | Feeling safe × context | 0.529 | 0.199 | 0.140 | 0.918 | 0.008 | **0.036** |

*Note.* FDR = false discovery rate, SE = standard error.

^a^ Metric invariance was supported across sex.

^b^ Metric invariance could not be tested because there were too few cases in one of the categories for one of the sexes

**Table S5.** **Regression models of the association between individual-level constructs and mental health resilience adjusted for prenatal tobacco exposure in the Bavarian Longitudinal Study and prenatal tobacco and alcohol exposure in the Millennium Cohort Study.**

|  |  |  | Crude models | | | | Adjusted models | | | |
| --- | --- | --- | --- | --- | --- | --- | --- | --- | --- | --- |
| System level | Instrument – Age of assessment | Construct, subscale, or item | β | SE | 95% CI | | β | SE | 95% CI | |
| Bavarian Longitudinal Study | | | | | | | | | | |
| Individual | Emotionality, Activity, Sociability (EAS) – 6y | Self-regulation/temperament |  |  |  |  |  |  |  |  |
|  |  | Effortful control | -0.228 | 0.045 | -0.316 | -0.140 | -0.230 | 0.044 | -0.316 | -0.145 |
|  |  | Activity | 0.061 | 0.047 | -0.030 | 0.152 | 0.061 | 0.045 | -0.028 | 0.149 |
|  |  | Shyness | -0.041 | 0.054 | -0.148 | 0.065 | -0.043 | 0.053 | -0.148 | 0.061 |
|  |  | Emotionality | 0.257 | 0.048 | 0.162 | 0.351 | 0.257 | 0.047 | 0.166 | 0.349 |
|  |  | Sociability | -0.120 | 0.061 | -0.240 | -0.001 | -0.118 | 0.059 | -0.234 | -0.001 |
|  | Tester’s Rating of Child Behaviour (TRCB) – 6y | Social-emotional regulation | -0.030 | 0.047 | -0.126 | 0.060 | -0.032 | 0.048 | -0.126 | 0.061 |
|  | Team Rating of Child Attention (TEAM) – 6y | Attention | -0.181 | 0.050 | -0.279 | -0.082 | -0.181 | 0.049 | -0.277 | -0.085 |
|  | Harter scales (parent-rated) – 6y | Parent-perceived competence |  |  |  |  |  |  |  |  |
|  |  | Cognition | -0.119 | 0.055 | -0.227 | -0.011 | -0.120 | 0.054 | -0.227 | -0.014 |
|  |  | Peers | -0.050 | 0.051 | -0.150 | 0.049 | -0.049 | 0.051 | -0.148 | 0.051 |
|  |  | Motor | -0.140 | 0.052 | -0.242 | -0.038 | -0.140 | 0.051 | -0.241 | -0.039 |
|  |  | Maternal relationship | -0.058 | 0.048 | -0.152 | 0.035 | -0.062 | 0.048 | -0.155 | 0.032 |
|  | Kaufman Assessment Battery for Children (KABC) – 6y | Cognitive abilities | -0.154 | 0.053 | -0.258 | -0.051 | -0.154 | 0.052 | -0.256 | -0.053 |
|  | Social Inhibition Assessment – 6y | Social inhibition | -0.046 | 0.048 | -0.141 | 0.049 | -0.046 | 0.047 | -0.139 | 0.046 |
| Millennium Cohort Study | | | | | | | | | | |
| Individual | Child Social Behaviour Questionnaire (CSBQ) – 5y | Independence and self-regulation | -0.158 | 0.040 | -0.237 | -0.079 | -0.163 | 0.038 | -0.238 | -0.088 |
|  |  | Emotional dysregulation | 0.494 | 0.032 | 0.432 | 0.556 | 0.450 | 0.032 | 0.388 | 0.512 |
|  | British Ability Scales (BAS) – 5y | Naming Vocabulary | -0.106 | 0.042 | -0.188 | -0.025 | -0.078 | 0.040 | -0.157 | 0.001 |
|  |  | Picture Similarities | -0.020 | 0.049 | -0.116 | 0.076 | -0.010 | 0.046 | -0.101 | 0.081 |
|  |  | Pattern Construction | -0.078 | 0.062 | -0.201 | 0.044 | -0.082 | 0.056 | -0.192 | 0.029 |

*Note*. CI = confidence interval, SE = standard error.

**Figure S3. Effects of sex (A, C) and contextual adversity (B, D) on mental health resilience and protective, promotive, and risk factors in the Bavarian Longitudinal Study (A, B) and the Millennium Cohort Study (C, D).**

***Note.* Colouring is based on adjusted *p*-values and indicate significant differences after correction for the false discovery rate.**

**
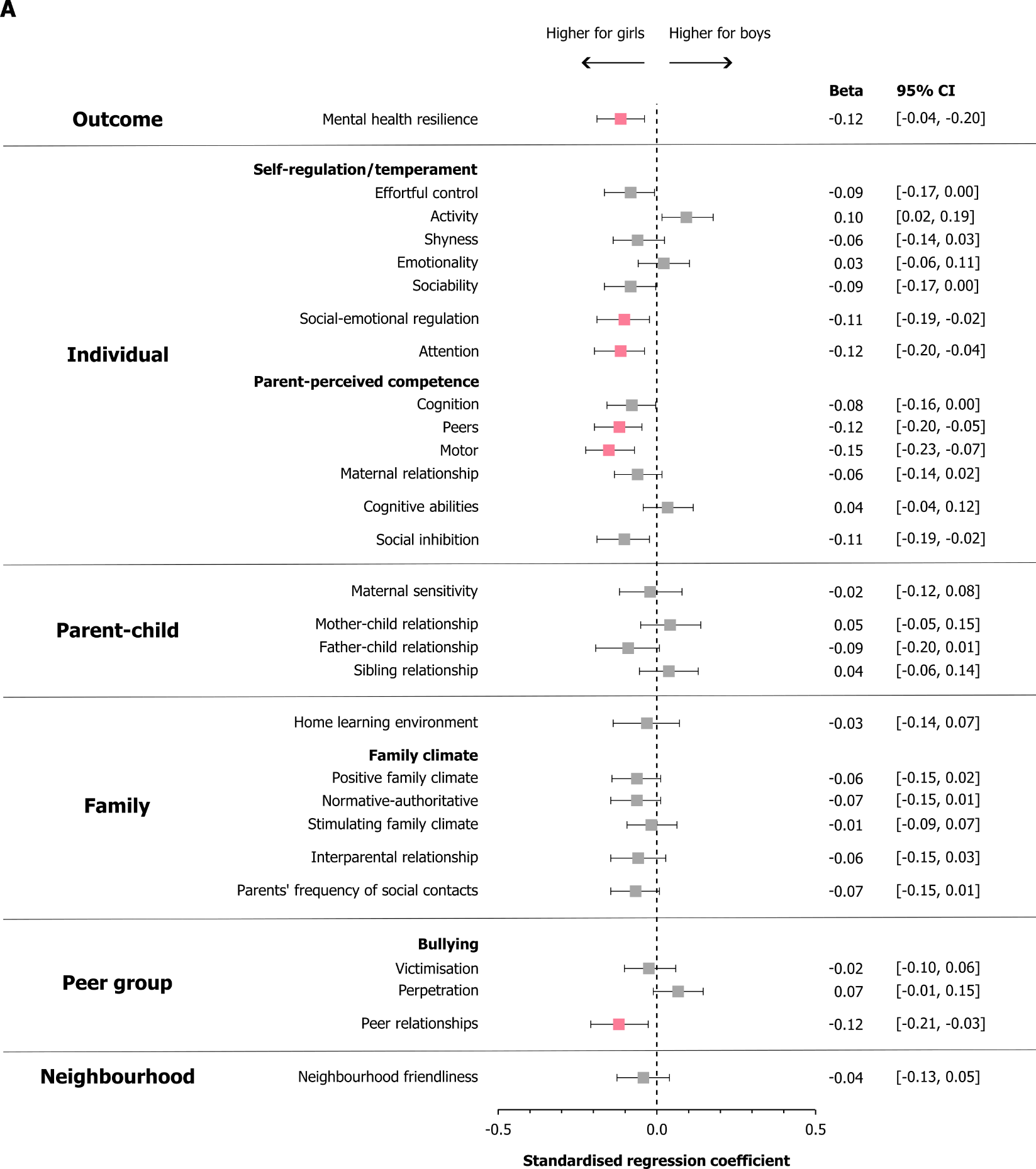
**


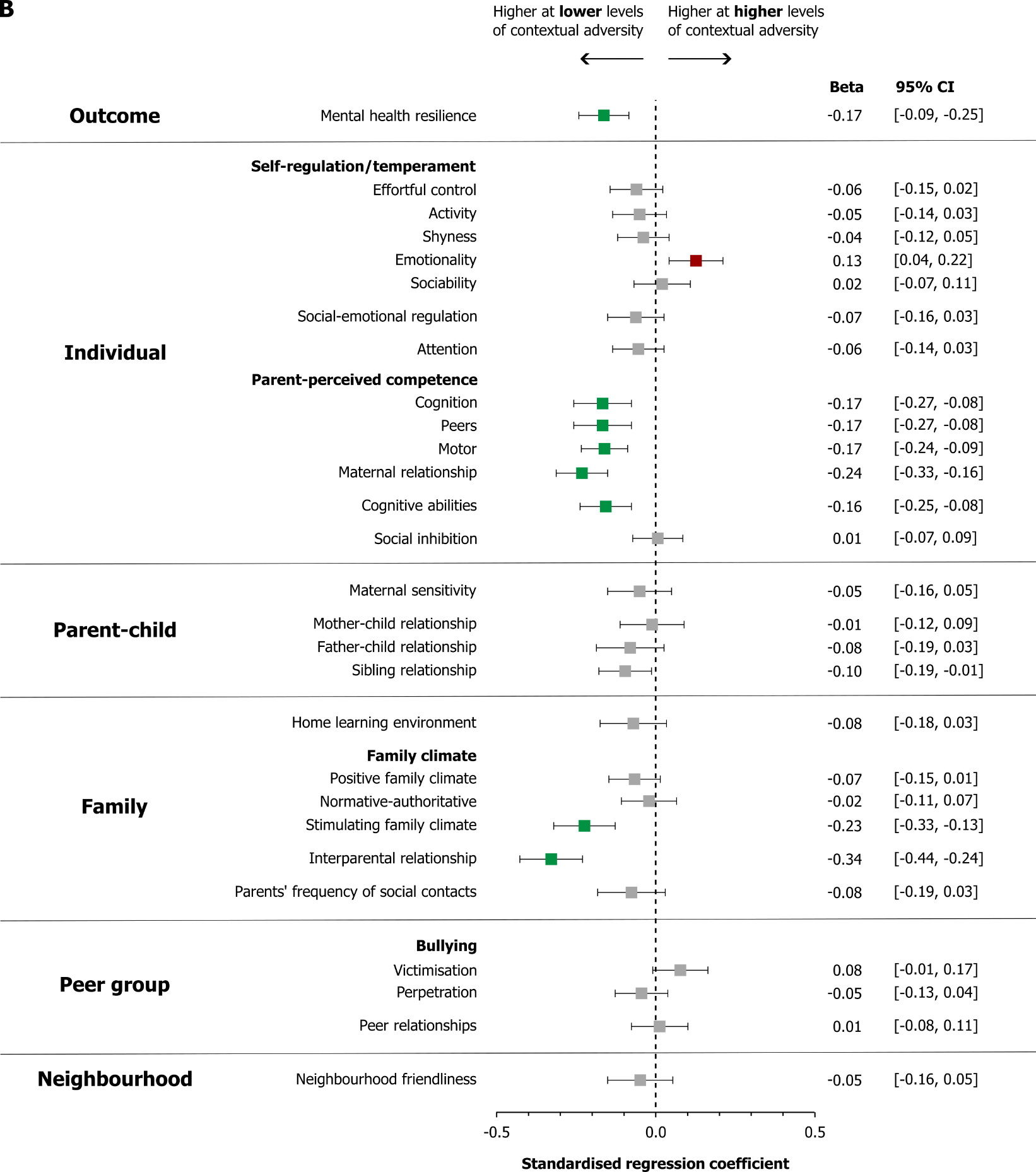


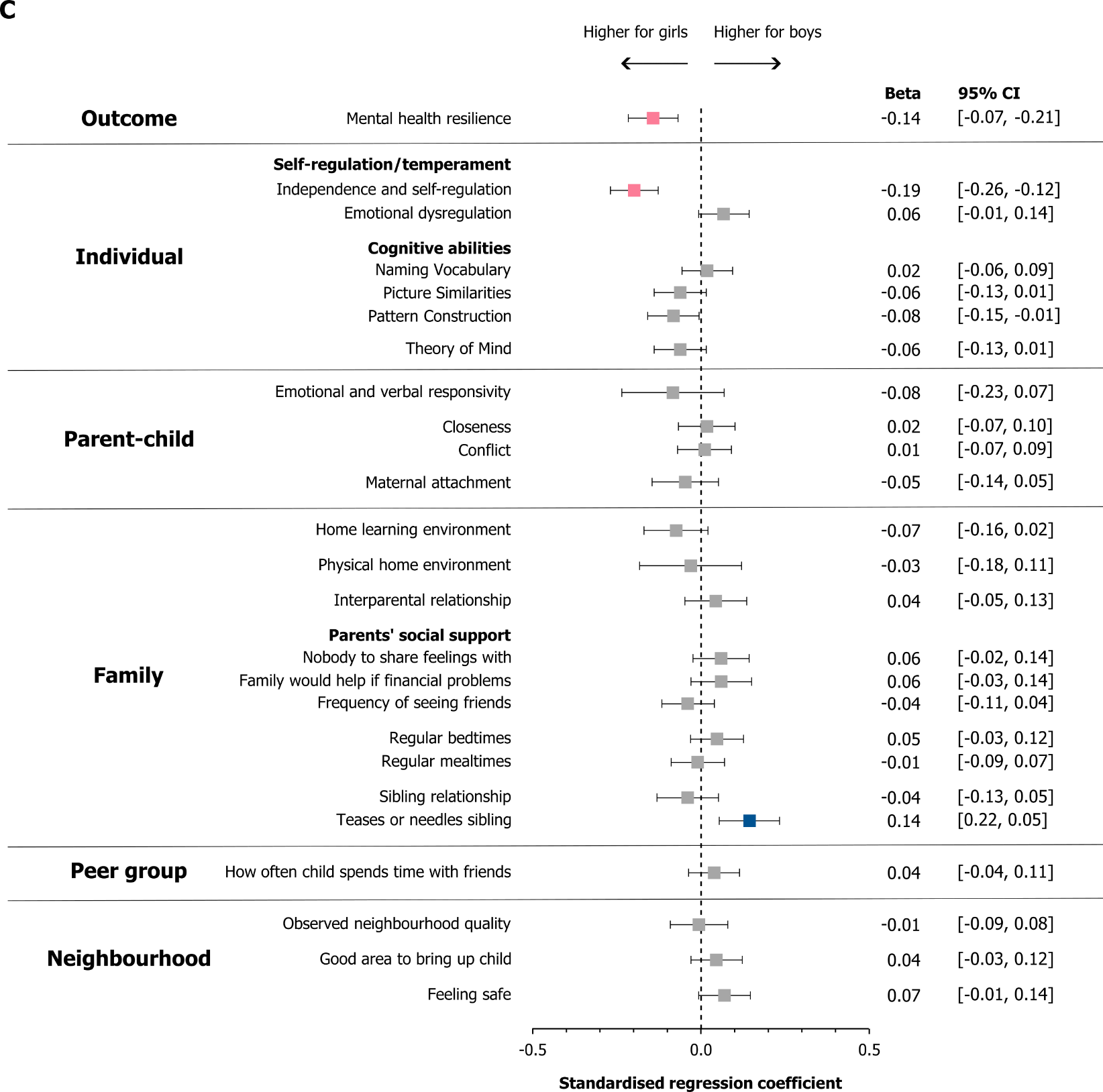


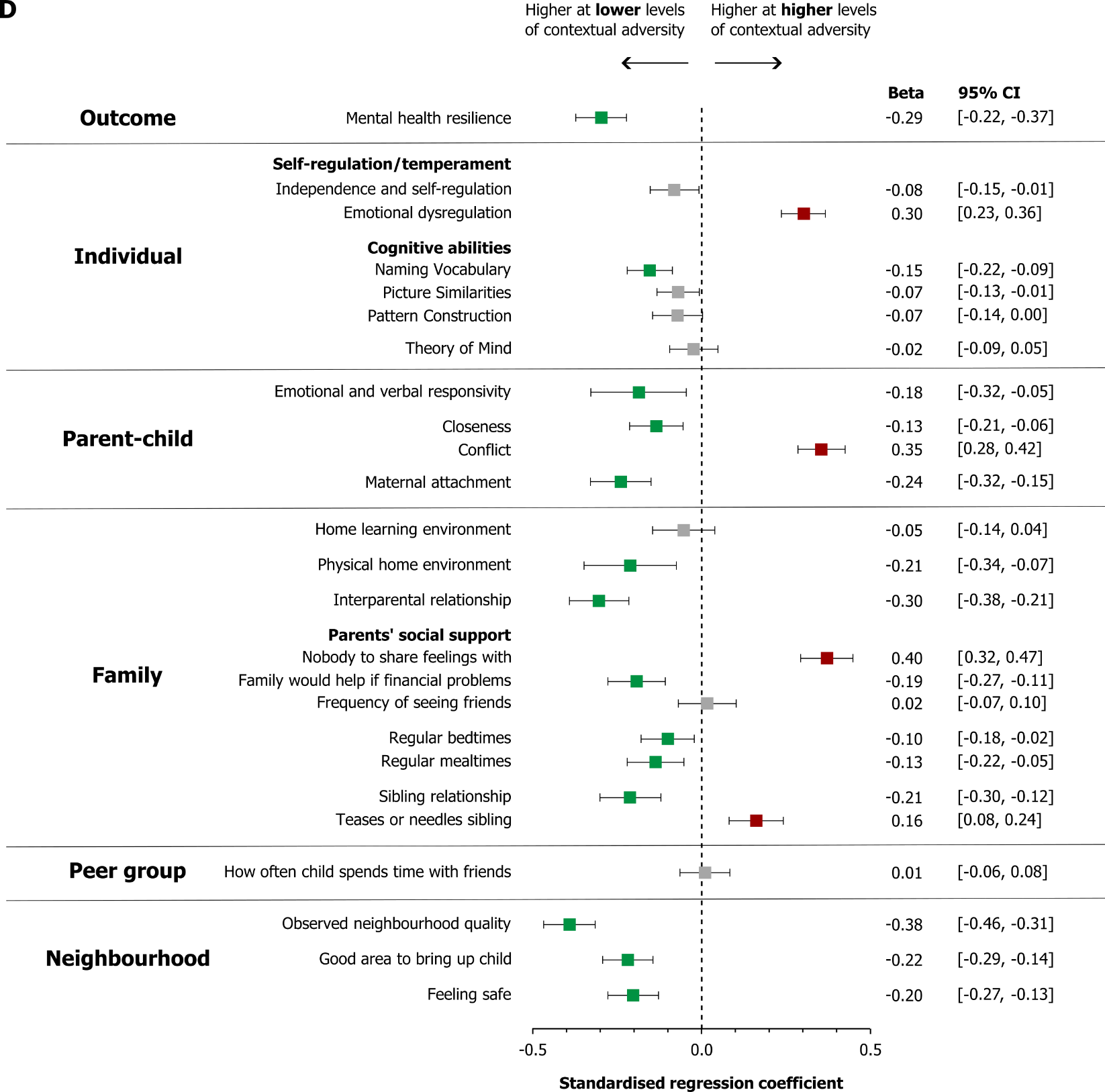


**Table S6. Indirect effects of sex and contextual adversity on mental health resilience through protective, promotive, and risk factors in the Bavarian Longitudinal Study.**

|  |  | Indirect - Sex | | | | | | Indirect - Contextual adversity | | | | | |
| --- | --- | --- | --- | --- | --- | --- | --- | --- | --- | --- | --- | --- | --- |
|  |  |  |  | bootstrap CI | |  |  |  |  | bootstrap CI | |  |  |
|  |  | β | SE | lower | upper | *p* | FDR-corrected *p* | β | SE | lower | upper | *p* | FDR-corrected *p* |
| Self-regulation/ temperament (EAS) | Effortful control | 0.019 | 0.009 | 0.002 | 0.038 | 0.033 | 0.111 | 0.013 | 0.009 | -0.004 | 0.033 | 0.134 | 0.283 |
|  | Activity | 0.006 | 0.006 | -0.003 | 0.019 | 0.234 | 0.401 | -0.003 | 0.004 | -0.013 | 0.002 | 0.384 | 0.523 |
|  | Shyness | 0.001 | 0.004 | -0.005 | 0.010 | 0.712 | 0.732 | 0.001 | 0.003 | -0.005 | 0.009 | 0.742 | 0.746 |
|  | Emotionality | 0.006 | 0.010 | -0.013 | 0.027 | 0.575 | 0.651 | 0.031 | 0.012 | 0.011 | 0.057 | 0.003 | **0.017** |
|  | Sociability | 0.009 | 0.007 | -0.001 | 0.025 | 0.114 | 0.251 | -0.002 | 0.006 | -0.016 | 0.008 | 0.687 | 0.719 |
| Tester’s Rating of Child Behaviour (TRCB) | Social-emotional regulation | 0.001 | 0.005 | -0.009 | 0.014 | 0.759 | 0.750 | 0.001 | 0.003 | -0.006 | 0.009 | 0.789 | 0.758 |
| Team Rating of Child Attention (TEAM) | Attention | 0.018 | 0.008 | 0.004 | 0.037 | 0.006 | **0.029** | 0.008 | 0.007 | -0.004 | 0.023 | 0.192 | 0.349 |
| Perceived competence (Harter Scales),  parent-rated | Cognition | 0.009 | 0.007 | -0.001 | 0.026 | 0.080 | 0.209 | 0.020 | 0.012 | 0.001 | 0.048 | 0.034 | 0.112 |
|  | Peers | 0.005 | 0.007 | -0.008 | 0.019 | 0.430 | 0.555 | 0.006 | 0.009 | -0.012 | 0.025 | 0.430 | 0.555 |
|  | Motor | 0.017 | 0.009 | 0.001 | 0.038 | 0.044 | 0.136 | 0.019 | 0.010 | 0.002 | 0.042 | 0.044 | 0.136 |
|  | Maternal relationship | 0.002 | 0.004 | -0.003 | 0.012 | 0.514 | 0.615 | 0.009 | 0.012 | -0.014 | 0.034 | 0.447 | 0.567 |
| Kaufman Assessment Battery for Children (KABC) | Cognitive abilities | -0.005 | 0.006 | -0.020 | 0.005 | 0.350 | 0.496 | 0.021 | 0.010 | 0.003 | 0.042 | 0.017 | 0.069 |
| Social Inhibition Assessment | Social inhibition | 0.004 | 0.006 | -0.008 | 0.015 | 0.460 | 0.575 | 0.000 | 0.003 | -0.006 | 0.005 | 0.931 | 0.823 |
| AMCIES | Maternal sensitivity | 0.007 | 0.008 | -0.005 | 0.025 | 0.279 | 0.443 | 0.006 | 0.007 | -0.008 | 0.021 | 0.438 | 0.560 |
| Card-Sorting Task | Mother-child relationship | -0.006 | 0.007 | -0.022 | 0.007 | 0.382 | 0.523 | 0.002 | 0.007 | -0.013 | 0.017 | 0.778 | 0.758 |
|  | Father-child relationship | 0.004 | 0.006 | -0.005 | 0.019 | 0.408 | 0.543 | 0.004 | 0.006 | -0.004 | 0.020 | 0.435 | 0.558 |
|  | Sibling relationship | 0.003 | 0.005 | -0.005 | 0.015 | 0.413 | 0.548 | -0.007 | 0.007 | -0.024 | 0.003 | 0.181 | 0.340 |
| Home Observation for Measurement of the Environment (HOME) | Home learning environment | 0.001 | 0.004 | -0.007 | 0.012 | 0.805 | 0.759 | 0.002 | 0.005 | -0.004 | 0.018 | 0.583 | 0.657 |
| Family climate (FES) | Positive family climate | 0.004 | -0.013 | 0.003 | 0.312 | 0.477 | 0.004 | -0.004 | 0.004 | -0.015 | 0.002 | 0.244 | 0.405 |
|  | Normative-authoritative | 0.006 | -0.002 | 0.022 | 0.135 | 0.284 | 0.006 | 0.003 | 0.005 | -0.007 | 0.014 | 0.654 | 0.712 |
|  | Stimulating family climate | 0.003 | -0.006 | 0.008 | 0.852 | 0.783 | 0.003 | 0.013 | 0.011 | -0.006 | 0.036 | 0.197 | 0.353 |
| Dyadic Adjustment Scale (DAS) | Interparental relationship | 0.013 | 0.010 | -0.004 | 0.033 | 0.152 | 0.305 | 0.072 | 0.020 | 0.034 | 0.115 | 0.000 | **0.000** |
| Parents' social support | Parents' frequency of social contacts | 0.002 | 0.004 | -0.005 | 0.012 | 0.523 | 0.619 | 0.003 | 0.004 | -0.005 | 0.012 | 0.531 | 0.623 |
| Bullying | Perpetration | 0.010 | 0.007 | -0.001 | 0.025 | 0.093 | 0.226 | -0.007 | 0.007 | -0.023 | 0.006 | 0.247 | 0.405 |
|  | Victimisation | -0.004 | 0.008 | -0.022 | 0.011 | 0.610 | 0.681 | 0.014 | 0.009 | -0.001 | 0.032 | 0.088 | 0.223 |
| Card-Sorting Task | Peer relationships | 0.010 | 0.007 | -0.002 | 0.026 | 0.104 | 0.240 | 0.000 | 0.004 | -0.010 | 0.009 | 0.865 | 0.785 |
| Neighbourhood | Child friendliness neighbourhood | 0.004 | 0.005 | -0.005 | 0.015 | 0.387 | 0.525 | 0.005 | 0.005 | -0.004 | 0.016 | 0.307 | 0.471 |

*Note.* AMCIES = Assessment of Mother-Child-Interactions with the Etch-a-Sketch, CI = confidence interval, FDR = false discovery rate, SE = standard error.

**Table S7. Indirect effects of sex and contextual adversity on mental health resilience through protective, promotive, and risk factors in the Millennium Cohort Study.**

|  |  | Indirect - Sex | | | | | | Indirect - Contextual adversity | | | | | | |
| --- | --- | --- | --- | --- | --- | --- | --- | --- | --- | --- | --- | --- | --- | --- |
|  |  |  |  | bootstrap CI | |  |  |  |  | bootstrap CI | |  |  |  |
|  |  | β | SE | lower | upper | *p* | FDR-corrected *p* | β | SE | lower | upper | *p* | FDR-corrected *p* |  |
| Child Social Behaviour Questionnaire (CSBQ) | Independence and self-regulation | 0.028 | 0.010 | 0.011 | 0.048 | 0.000 | **0.000** | 0.012 | 0.007 | 0.001 | 0.026 | 0.032 | 0.109 |  |
|  | Emotional dysregulation | 0.027 | 0.017 | -0.008 | 0.060 | 0.113 | 0.251 | 0.132 | 0.019 | 0.097 | 0.168 | 0.000 | **0.000** |  |
| British Ability Scales (BAS) | Naming Vocabulary | -0.001 | 0.003 | -0.009 | 0.005 | 0.684 | 0.718 | 0.012 | 0.007 | 0.000 | 0.026 | 0.050 | 0.147 |  |
|  | Picture Similarities | 0.001 | 0.003 | -0.006 | 0.009 | 0.815 | 0.763 | 0.001 | 0.004 | -0.006 | 0.009 | 0.800 | 0.758 |  |
|  | Pattern Construction | 0.005 | 0.006 | -0.004 | 0.018 | 0.334 | 0.495 | 0.004 | 0.006 | -0.003 | 0.018 | 0.346 | 0.495 |  |
| Home Observation for Measurement of the Environment (HOME) | Emotional and verbal responsivity | 0.011 | 0.012 | -0.007 | 0.040 | 0.224 | 0.389 | 0.023 | 0.016 | 0.002 | 0.065 | 0.023 | 0.086 |  |
| Child-Parent Relationship Scale (CPRS) | Closeness subscale | -0.002 | 0.005 | -0.012 | 0.010 | 0.711 | 0.732 | 0.016 | 0.010 | 0.002 | 0.041 | 0.015 | 0.062 |  |
|  | Conflict subscale | 0.002 | 0.009 | -0.017 | 0.020 | 0.889 | 0.798 | 0.079 | 0.021 | 0.036 | 0.121 | 0.000 | **0.000** |  |
| Condon questionnaire | Maternal attachment | 0.008 | 0.008 | -0.004 | 0.028 | 0.247 | 0.405 | 0.032 | 0.016 | 0.004 | 0.067 | 0.025 | 0.092 |  |
| HOME | Home learning environment | 0.004 | 0.006 | -0.006 | 0.019 | 0.513 | 0.615 | 0.004 | 0.006 | -0.006 | 0.018 | 0.424 | 0.555 |  |
| HOME | Physical home environment | 0.006 | 0.009 | -0.014 | 0.027 | 0.462 | 0.575 | 0.025 | 0.013 | 0.005 | 0.053 | 0.006 | **0.029** |  |
| Golombok Rust Inventory of Marital State | Interparental relationship quality | -0.004 | 0.006 | -0.018 | 0.005 | 0.415 | 0.548 | 0.030 | 0.022 | -0.010 | 0.076 | 0.128 | 0.272 |  |
| Parents' social support, parent interview | Nobody to share feelings with | -0.002 | 0.004 | -0.012 | 0.007 | 0.678 | 0.718 | 0.012 | 0.024 | -0.030 | 0.061 | 0.576 | 0.651 |  |
|  | Family would help if financial problems | -0.003 | 0.005 | -0.015 | 0.005 | 0.455 | 0.573 | 0.012 | 0.012 | -0.012 | 0.037 | 0.278 | 0.443 |  |
|  | Frequency of seeing friends | 0.001 | 0.003 | -0.003 | 0.008 | 0.547 | 0.633 | -0.001 | 0.003 | -0.008 | 0.004 | 0.735 | 0.744 |  |
| Family routines,  parent interview | Regular bedtimes | -0.007 | 0.007 | -0.022 | 0.005 | 0.258 | 0.419 | 0.015 | 0.008 | 0.002 | 0.033 | 0.018 | 0.071 |  |
|  | Regular mealtimes | 0.000 | 0.002 | -0.004 | 0.006 | 0.896 | 0.802 | 0.003 | 0.008 | -0.014 | 0.020 | 0.791 | 0.758 |  |
| Sibling relationship, parent interview | Sibling relationship | 0.005 | 0.007 | -0.007 | 0.021 | 0.455 | 0.573 | 0.030 | 0.013 | 0.007 | 0.060 | 0.006 | **0.029** |  |
|  | Teases or needles sibling | 0.018 | 0.009 | 0.004 | 0.039 | 0.006 | **0.029** | 0.021 | 0.009 | 0.006 | 0.041 | 0.003 | **0.017** |  |
| Friendships, parent interview | How often child spend time with friends | 0.003 | 0.004 | -0.003 | 0.012 | 0.374 | 0.517 | 0.001 | 0.003 | -0.006 | 0.009 | 0.761 | 0.750 |  |
| Neighbourhood | Observed neighbourhood quality | 0.000 | 0.007 | -0.014 | 0.014 | 0.984 | 0.851 | 0.065 | 0.020 | 0.023 | 0.108 | 0.003 | **0.017** |  |
|  | Good area to bring up a child | -0.004 | 0.005 | -0.016 | 0.004 | 0.253 | 0.413 | 0.022 | 0.011 | 0.002 | 0.046 | 0.023 | 0.086 |  |
|  | Feeling safe | 0.002 | 0.004 | -0.004 | 0.010 | 0.531 | 0.623 | -0.007 | 0.010 | -0.026 | 0.011 | 0.486 | 0.592 |  |

*Note.* CI = confidence interval, FDR = false discovery rate, SE = standard error.
